# Empirical *in operando* analysis of the charge carrier dynamics in hematite photoanodes by PEIS, IMPS and IMVS†
†Electronic supplementary information (ESI) available. See DOI: 10.1039/c6cp04683e



**DOI:** 10.1039/c6cp04683e

**Published:** 2016-08-01

**Authors:** Dino Klotz, David Shai Ellis, Hen Dotan, Avner Rothschild

**Affiliations:** a Department of Materials Science and Engineering , Technion – Israel Institute of Technology , 3200003 Haifa , Israel . Email: dino@technion.ac.il ; Email: avner@mt.technion.ac.il; b Institute for Applied Materials – Materials for Electrical and Electronic Engineering (IAM-WET) , Karlsruhe Institute of Technology (KIT) , 76131 Karlsruhe , Germany

## Abstract

In this Perspective, we introduce intensity modulated photocurrent/voltage spectroscopy (IMPS and IMVS) as powerful tools for the analysis of charge carrier dynamics in photoelectrochemical (PEC) cells for solar water splitting, taking hematite (α-Fe_2_O_3_) photoanodes as a case study. We complete the picture by including photoelectrochemical impedance spectroscopy (PEIS) and linking the trio of PEIS, IMPS and IMVS, introduced here as photoelectrochemical immittance triplets (PIT), both mathematically and phenomenologically, demonstrating what conclusions can be extracted from these measurements. A novel way of analyzing the results by an empirical approach with minimal presumptions is introduced, using the distribution of relaxation times (DRT) function. The DRT approach is compared to conventional analysis approaches that are based on physical models and therefore come with model presumptions. This work uses a thin film hematite photoanode as a model system, but the approach can be applied to other PEC systems as well.

## Abstract 2

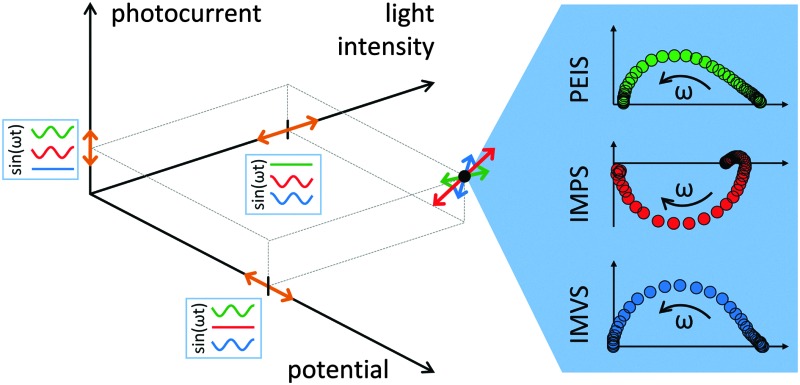
We introduce an empirical *in operando* analysis of the charge carrier dynamics in hematite photoanodes by PEIS, IMPS and IMVS.

## 1. Introduction

### 1.1. Photoelectrochemical water splitting

Photoelectrochemical water splitting by photoelectrochemical (PEC) cells has attracted a lot of attention during the last decade. A large variety of new approaches and concepts have shown promising progress towards improving solar-to-hydrogen (STH) efficiencies. These may lead to an economically efficient use of solar water splitting as a technique to provide hydrogen for a sustainable energy future.^1,2^ Along with these developments, a host of measurement results are presented and the related techniques are constantly being developed.

The key part of a PEC cell is the photoelectrode, because it constitutes the bottleneck for efficient photoelectrolysis.^3^ Photoelectrochemical measurements on photoelectrodes fulfill three basic purposes. Firstly, they provide the basis for benchmarking different types of electrodes.^4^ Secondly, they provide diagnostics to identify the elementary reaction steps that take place during the operation of a photoelectrode, and measure their kinetics.^5–7^ And thirdly, they quantify different losses within the photoelectrode or at the photoelectrode/electrolyte interface,^8^ thereby identifying the processes that limit the photoelectrode performance. Even though there is a lot of ongoing work in the field, surprisingly little is known about the rate-determining steps and the related reactions.^9^ However, this knowledge is essential to guide the efforts to improve the photoelectrode efficiency. The current status of modeling the underlying processes such as charge carrier generation, recombination and transfer to the liquid electrolyte was recently reviewed and classified in ref. 10.

Hematite (α-Fe_2_O_3_) is a promising candidate for use as a photoanode in PEC cells.^11,12^ It is stable in aqueous alkaline solutions, has suitable bandgap energy for sunlight harvesting, and is an earth-abundant low-cost material. Especially their long term stability^13^ make hematite photoanodes an excellent choice for a study that requires stable and reproducible measurements. Challenges to overcome are the poor charge carrier mobility^14^ and the short lifetime of photogenerated charge carriers^15^ that lead to significant bulk recombination and short charge collection length.^16^ The model system we consider here is a thin film hematite photoanode on fluorine-doped tin oxide (FTO) coated glass substrate, measured in room temperature with 1 M NaOH in deionized water as an electrolyte. Details about the cell and the test setup can be found in the ESI,† S1 and in ref. 8.

### 1.2. Photoelectrochemical measurement techniques

Performance of photoelectrodes is usually characterized by photocurrent–potential (*J*–*U*) curves‡
‡Common other denominations of *J*–*U* curves are: *J*–*V* curves, *I*–*V* curves, *I*–*E* characteristics, CV curves or scans (for cyclic-voltammetry) or cyclovoltammograms, linear sweep voltammograms (LSV), cycloamperographs and possibly even more.
^ ^^4,11,17^ under constant illumination. The net photocurrent density, *J*, is directly proportional to the rate of water photoelectrolysis when the Faradaic efficiency is 100%, which is a viable assumption for hematite photoanodes.^18^ Therefore, a high photocurrent density indicates good cell performance.^19,20^ Other useful techniques include chopped-light voltammetry^3,7,8,17,21^ and IPCE measurements.^21–23^ These methods are sufficient to test the performance and efficiency of the photoanode, but they do not provide detailed information about the reaction mechanisms and how these contribute to the losses or limit the performance. For benchmarking and analyzing the water photoelectrolysis performance, measurements must be carried out in aqueous electrolytes free of species that may undergo redox reactions that would compete with the water oxidation or reduction reactions. However, for diagnostic purposes, oxidizing or reducing agents may be added to the electrolyte solution in order to scrutinize different processes occurring within the photoelectrode and at the photoelectrode/electrolyte interface. For example, H_2_O_2_ can be added as a hole scavenger that gives rise to fast charge transfer kinetics at the photoelectrode/electrolyte interface, which allows decoupling and quantifying the different processes occurring within the photoelectrode and at the photoelectrode/electrolyte interface.^8^ However, since the electrochemical reactions at the surface of the photoelectrode may influence the surface charge, and consequently also the space charge properties (*e.g.*, space charge width and band bending), care must be taken to account for these effects when comparing measurements with or without additional agents, especially for hematite photoanodes which are thought to be sensitive to these properties.^24^ In order to gain a deeper understanding about the intrinsic properties of hematite as a semiconductor, Mott–Schottky experiments are well-established.^16^ They provide information about flat-band potential and the majority charge carrier concentration in equilibrium. That is essential information but it is not certain whether the Mott–Schottky relation, and the underlying model,^25^ is valid for thin films.^26^

Of the above mentioned measurements, only chopped-light voltammetry allows the possibility to probe the time dependent effects of changes in light intensity on the properties of the photoanode. Two of the few studies that investigated magnitude and area of the so-called spikes in chopped-light measurements are ref. 27 and 28. However, the time resolved model for surface charging developed in ref. 29 includes a distinct charging current component that is not in agreement with ref. 27 and 28. Surface charging can also be determined by “charging” the surface under illumination at high applied potential, and subsequently switching off the light and conducting a fast voltage scan towards lower potentials, as explained in ref. 7 and 30. Another promising approach is to analyze the absorption signal of the photoanode after a step in pump light excitation, and compare it to the photocurrent.^9,31–34^ The amount of charging has been related to the photocurrent in ref. 9, but the exact role of the charges is yet unclear and actively discussed in recent literature.^35^

Frequency domain techniques are able to distinguish between different photoelectrochemical processes by their respective time constants. Applying frequency domain techniques *in operando* for relevant input and output quantities further promises to probe the dynamics and system properties exactly as they appear during operation. Although characterized through frequency domain techniques, these processes determine the steady state operation. In that context, photoelectrochemical impedance spectroscopy (PEIS) is a powerful tool to investigate the dynamic relation between photovoltage and photocurrent.^3,5,6,36–40^ Results for PEIS have been used to clarify processes in many different electrochemical devices.^40^ The work of Klahr and co-workers^7,36,41,42^ was among the first to establish a comprehensive equivalent circuit model (ECM, also: equivalent circuit modeling) for hematite photoanodes. Other promising approaches were recently published.^17,43–45^

In photoelectrochemistry, there are three relevant effective parameters that determine the performance of a PEC device: potential, photocurrent and light intensity. The three possible configurations to probe their dynamic relations are represented by the trio of electrochemical impedance and optical modulation techniques (photoelectrochemical immittance^46^ techniques, see also Section 2):

• photoelectrochemical impedance spectroscopy (PEIS),

• intensity-modulated photocurrent spectroscopy (IMPS),

• intensity-modulated photovoltage spectroscopy (IMVS).

Their combined analysis offers the potential to gain access to elusive parameters that govern the water oxidation and reduction reactions on photoanodes or photocathodes, respectively. IMPS and IMVS are relatively uncommon techniques that probe the dynamic relation between irradiation and the electrochemical response of the photoelectrode. Much of the theory adopted herein has its foundations in ref. 38. Macdonald introduced the definition for IMPS in his seminal textbook on impedance spectroscopy.^40^ The field was significantly developed by the pioneering work of Peter and co-workers in the 90's,^29,38^ which includes a broad introduction to IMPS. The relation of PEIS and IMPS has been treated in ref. 39 and 47 for the hydrogen evolution on p-InP photocathodes. Schefold has presented a fundamental theoretical approach with a clear explanation of the relations between IMPS and PEIS.^39^

Despite the potential and relevance of the IMVS technique, there is, to the best of our knowledge, only one publication using the basic application and analysis of IMVS for hematite photoanodes.^48^ In contrast, IMVS has been widely used to probe the recombination characteristics in dye-sensitized solar cells (DSSC).^49–52^ For DSSC as well as for hematite,^48^ IMVS is applied for open circuit conditions,^50^ *i.e.* with *J* = 0. This operating point is very different from the usual operating point of photoanodes so internal properties like band bending, drift current and space charge are expected to differ considerably. That is why we instead prefer to conduct measurements in the relevant operation conditions for photoanodes.

Whereas it was common practice to measure IMPS and IMVS at two different operating points (short and open circuit conditions, respectively), it was Halme^51^ who first brought them together to one common operating point for the case of DSSC, and provided the definitions of the physical quantities for excitation and system response based on ref. 40. We will adapt his findings to carry out a case study of thin film hematite photoanodes, and measure PEIS, IMPS and IMVS at several operating points. The trio of PEIS, IMPS and IMVS is introduced herein as photoelectrochemical immittance triplets (PIT).

### 1.3. Empirical analysis approach

Here, we will present an empirical analysis of PIT. The advantage and aim of this approach is that it keeps to a bare minimum any presumptions which can prejudice the results. *J*–*U* curves are a good example. For their analysis, the empirical raw data is used directly, with no further data treatment, and the obtained values yield unambiguous results.

One of the problems of frequency domain techniques is that they yield non-intuitive results that require further processing in order to obtain meaningful information. Usually the analysis of frequency domain data (*i.e.* impedance data) involves a fit to a model that is often not sufficiently verified. Thus, conclusions derived from such complicated analysis remain questionable. Another issue is the degree of simplification as determined by the model assumptions. If these assumptions are not valid or overlook effects that were presumed to be of second order while in fact they are not, the results might contain systematic errors. Especially for uncommon measurement techniques (such as IMPS and IMVS) and complex systems (such as hematite photoanodes), an unbiased empirical analysis approach is required to foster a clear understanding of the system and its behavior.

That is why we will start from the untreated results and will not assume any model for the analysis. We will show how to extract useful information out of the raw data by empirical methods using the distribution of relaxation times (DRT) analysis. The latter is a powerful method to display immittance data with superior capability to separate different polarization processes in the frequency domain, as compared to Nyquist or Bode plots. Unlike ECM, DRT analysis does not require any *a priori* model assumptions and is therefore a truly empirical method.^53,54^ It has been applied successfully to solid oxide fuel cells^55,56^ and lithium ion batteries.^57^ To appreciate the difficulties that may be encountered when not adopting such an approach, consider the impedance study of Li-ion batteries in ref. 57. By using an empirical DRT-based approach, the authors found that the contact resistance between the cathode and the metallic current collector caused a significant share of the overall losses. Without the benefit of hindsight, a pre-determined model may have easily overlooked this contribution to the loss, and a true deconvolution of the physical effects in the measurement may have been hindered. Suppose, hypothetically, that in our system a bad contact between the FTO transparent electrode (serving as a current collector) and hematite was likewise responsible for a large polarization resistance. A simple model based on charge carrier generation, separation, recombination and transfer alone would never be able to account for this phenomenon and, consequently, would never accurately represent the behavior of the photoanode for various operation points.

Here, we will describe how a DRT analysis together with basic mathematical operations can be used to identify the negative recombination current in hematite photoanodes,^31^ and show that the slow polarization process observed in the PEIS of hematite photoanodes below the onset potential can be related to this recombination current. While this result has already been suggested in the literature,^6,47^ a convincing proof for this interpretation has not been presented until now.

The measurements for our analysis are all conducted in a “cappuccino cell”^58^ without any sacrificial or redox agents in the alkaline aqueous electrolyte. Thus it is strictly an *in operando* characterization method that probes the underlying processes as they occur under the exact operation conditions of the photoelectrode. As for EIS, all PIT measurements are non-destructive. In view of these attributes, they have a great potential for broad application in the study of hematite photoanodes as well as in the field of photoelectrochemistry in general.

### 1.4. Paper structure overview

After the general introduction, Section 2 explains the measurement techniques (PEIS, IMPS and IMVS), and their experimental relationship in the photoelectrochemical immittance triplets (PIT) is highlighted. Section 3 summarizes three approaches for analyzing immittance spectra. Our empirical approach is introduced in detail in Section 4, followed by a discussion on the interpretation of the results and the conclusions that can be derived from them. While the whole paper is of considerable length, we have tried to keep the individual sections as self-contained as possible in order to provide a quick guide to the respective topics.

## 2. Photoelectrochemical immittance techniques

In this section, the measurement techniques used for our analysis approach will be presented. First, a three-dimensional diagram is introduced to display the static photocurrent density *J*, which depends on both potential, *U*, and light intensity, *I*. A brief introduction of the trio immittance (short for impedance and admittance^46^) techniques PEIS, IMPS and IMVS will be given, including treatment of raw data, and the issue of data quality is discussed. A more general introduction to immittance is provided in the ESI,† S3.

### 2.1. Representation of the static photocurrent density

**Fig. 1:**
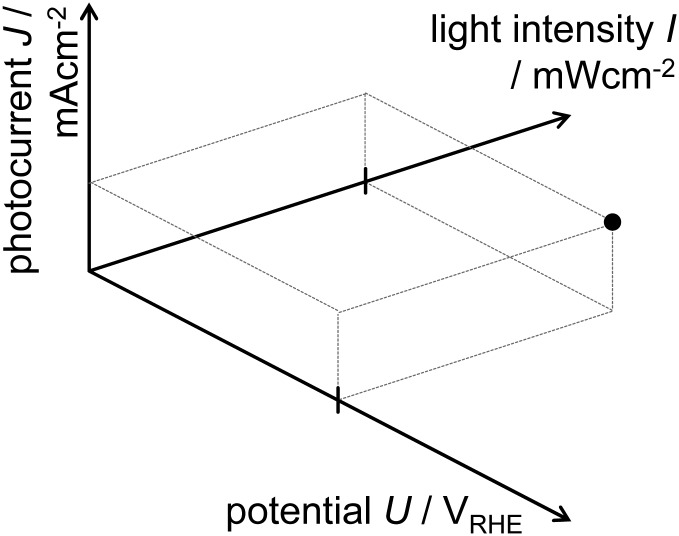
Static operating point of a photoanode following eqn (1).

The static operating point of a photoanode is defined as follows (see Fig. 1):

1 *J* = *f*(*U*,*I*),

where the current density, *J*, given in mA cm^–2^, is written as a function *f* of the bias potential, *U*, which is applied by the potentiostat and has units of V_RHE_ (volts against the reversible hydrogen electrode), and also of light intensity, *I*, given in mW cm^–2^. Both *I*, or alternatively the photon flux, *Φ*,^40^ (in photons per (cm^2^ s)) are common physical quantities to account for the magnitude of illumination applied to the cell. We use the light intensity (*I*) throughout this study, as it is mostly done in recent publications on PEIS and IMPS on hematite photoanodes.^6,48^ A detailed comparison of physical quantities to account for the magnitude of the illumination is featured in the ESI,† S2.

**Fig. 2:**
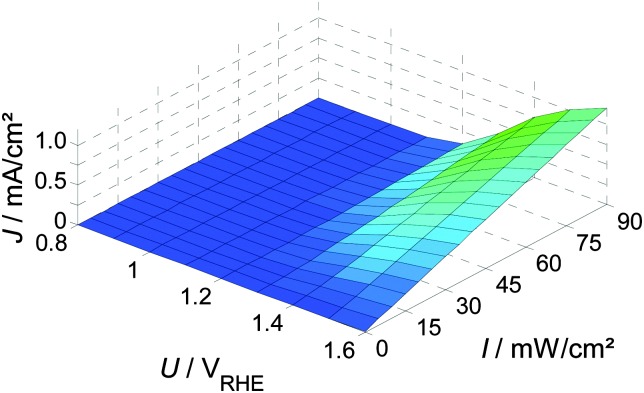
Measured static parameter map showing *J* and its dependency on *U* and *I* (for details about the experimental setup see ESI,† S1).

**Fig. 3:**
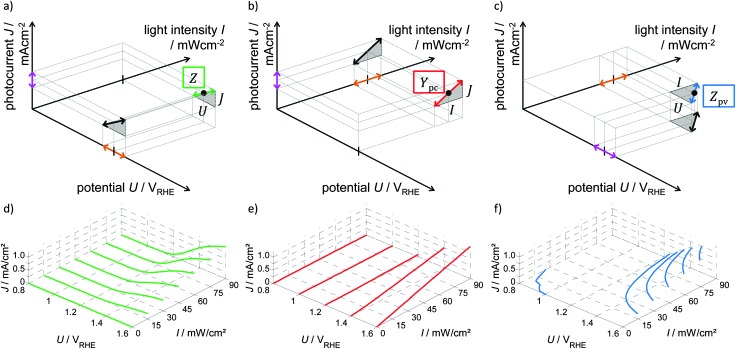
Basic relation between the potential, *U*, light intensity, *I*, and photocurrent, *J*, in the same three-dimensional representation as shown in Fig. 1 for (a) PEIS, (b) IMPS and (c) IMVS. The sinusoidal excitation is shown by orange arrows, the measured signal by pink arrows. The gradients (DC case) at a measurement point are indicated by double arrows. This gradient corresponds to the slope in the static (d) *J*–*U* curves, (e) *J*–*I* curves and (f) *U*–*I* curves.

When combining static *J*–*U* curves measured at different light intensities, a three dimensional parameter map is obtained, as shown in Fig. 2. Adding a third dimension to the display will be helpful for the introduction of the PEIS, IMPS and IMVS in the following sections. The slopes (*i.e.* gradients) in this display are directly connected to the values of the three techniques for very low frequencies (*ω* → 0), as can be seen in Fig. 3 (below).

### 2.2. Photoelectrochemical impedance spectroscopy (PEIS)

In PEIS, the impedance, *Z*(*ω*), of the photoanode is measured as a function of the angular frequency, *ω*. For clarification that the sample was illuminated during measurement, the “P” is inserted in the name of this technique. There is no other difference from conventional EIS. A time dependent part in *U*(*t*) is usually applied as small signal excitation, and *J*(*t*) is measured as the response signal. This mode is called potentiostatic excitation and it is also the recommended excitation mode for photoanodes because of the relatively high resistance values.^54^ However, it is also possible to excite the cell with *J*(*t*) and measure *U*(*t*). Both stimulus and response signals have the general form

2 *S*(*t*) = *S*_0_ + *ŝ*·sin(*ωt* + *φ*_S_),

with *S*_0_ being the static bias value, *ŝ* being the small signal amplitude of the sinusoidal alternating signal with an angular frequency *ω* and phase angle *φ*_S_. PEIS measures the complex electrical impedance *Z*(*ω*) of the sample at the constant operating point given by eqn (1) (see also ESI,† S3):3
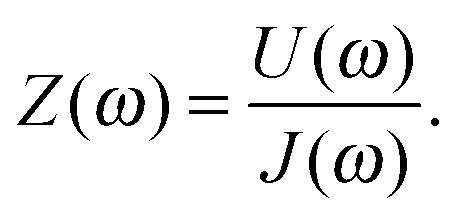
As *ω* → 0, the ratio of the respective small signal components of *J* and *U* determine the slope of the DC *J*–*U* curve shown in Fig. 3. We note that throughout this paper, we will refer to this and respective quantities for IMPS and IMVS as a “measured DC” value. Although the small signal response at exactly zero frequency is not directly measured, the measurement at the lowest frequency yields a very good estimate for this value (see the ESI,† S3). Fig. 3 also indicates the excitation signal (orange arrows), the measured signal (pink arrows) and the fixed light intensity. The green curves in Fig. 3d exemplarily demonstrate *J*–*U* curves measured at different light intensities. The slope of the these curves correspond to 1/*Z*(0) for the respective *U* and *I*, which is shown by the green arrow in Fig. 3a (at a given set of *U* and *I* values). The measured values are the same as in Fig. 2. It is worth mentioning that a well performing photoanode features a small DC impedance (at *ω* = 0), *Z*(0).

**Fig. 4:**
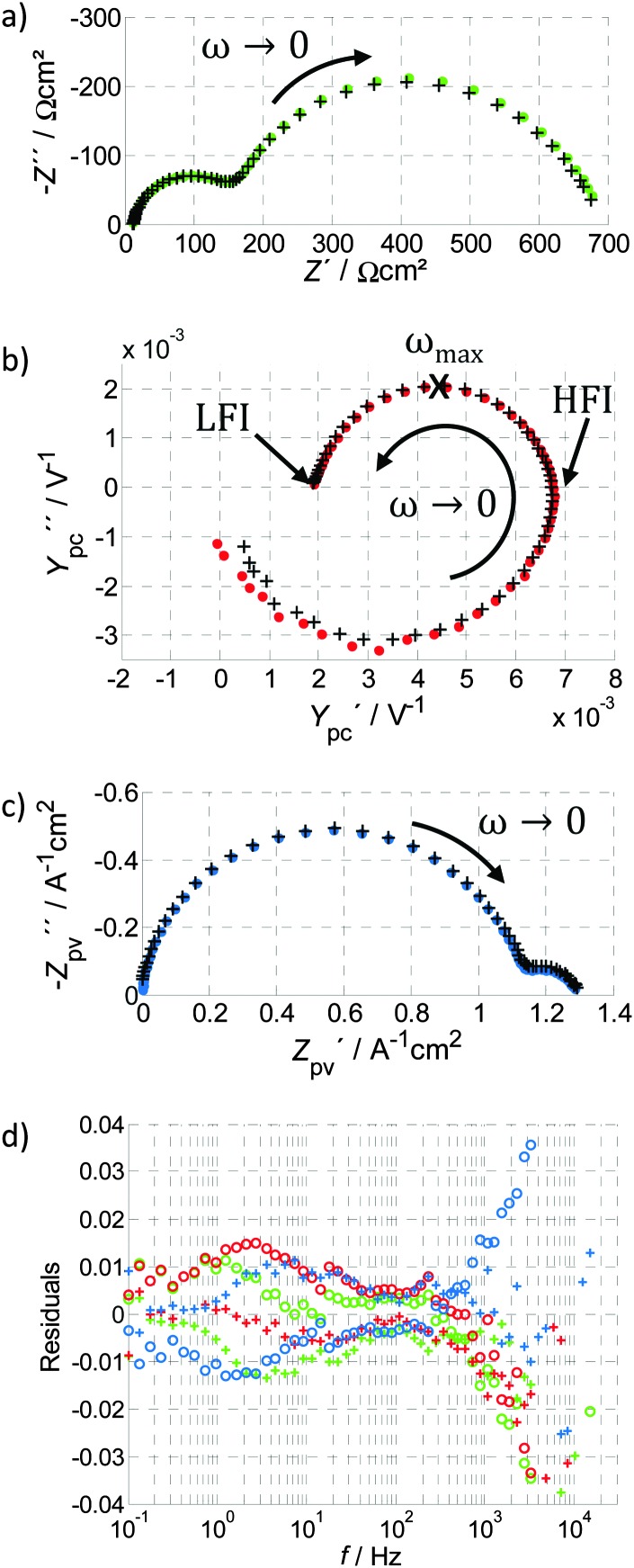
Examples for (a) PEIS, (b) IMPS, and (c) IMVS spectra, all measured at 1.22 V_RHE_ and 75 mW cm^–2^ from 22 kHz to 100 mHz. Circles mark the measurement results, while plus signs represent calculated values from the two complimentary measurements of the PIT, as discussed in Section 2.5. (d) Depicts the residuals for the real part (circles) and the imaginary part (plus signs) between measured and calculated results, presented in the respective colors (green for PEIS, red for IMPS and blue for IMVS). Extrapolation of the measured IMPS yields a *Y*_pc_(*ω* → ∞) of –3.0 × 10^–4^ V^–1^, whereas the calculated one yields *Y*_pc_(*ω* → ∞) of 7.3 × 10^–6^ V^–1^. This discrepancy is discussed in Section 2.5.

An impedance spectrum is usually displayed as a Nyquist plot as shown in Fig. 4a (below), and exhibits characteristic semicircles which are usually attributed to different polarization processes. Since the current is normalized by the area (*J* is a current density), the unit for *Z*(*ω*) is Ω cm^2^. The work of Klahr *et al.* represents the largest collection of PEIS measurements of hematite photoanodes, including an extensive parameter variation and the development of an ECM,^42^ which has been expanded in ref. 7 and 41. Other important studies for the field of hematite photoanodes include ref. 9, 17 and 43. More details about the impedance measurements are provided in the ESI,† S3. A complete introduction into EIS is given in ref. 40.

### 2.3. Intensity modulated photocurrent spectroscopy (IMPS)

IMPS is an advanced but still rarely used technique. The most detailed introduction to IMPS can be found in the fundamental work by Peter and co-workers.^29,38^ For IMPS, the excitation signal consists of the so-called background or bias light intensity, *I*_0_, plus the modulated sinusoidal signal with the amplitude, *Î*, both given as densities per cm^2^ and following the form of eqn (2). The potential, *U*, is fixed by the potentiostat and *J*(*t*) is measured as shown schematically by the arrows in Fig. 3b. The result of IMPS measurements is the frequency dependent photocurrent admittance, *Y*_pc_(*ω*):4
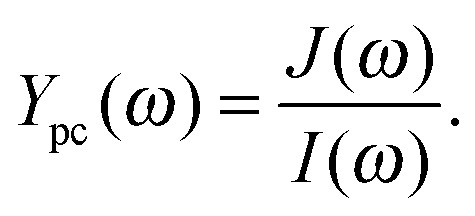

*Y*_pc_(*ω*) is measured in units of A cm^–2^ (W cm^–2^)^–1^, therefore its unit is V^–1^. The label photocurrent admittance indicates its relation to:

• excitation by a dynamic variation of the light intensity (*photo*current),

• response of the photocurrent density (photo*current*).

There are a number of definitions for IMPS to be found in the literature. The one suggested here is based on ref. 40 where the authors refer to it as IMPS admittance with the difference that we use the light intensity, *I*, instead of the normalized photon flux, *Φ*. *Y*_pc_(*ω*) is often referred to as *H*(*ω*),^3,37–39,59^ but there is no general agreement for the nomenclature. In the earlier definition of IMPS by Peter and Vanmaekelbergh, *Y*_pc_(*ω*) was called “photocurrent efficiency”.^29^ Later publications referred to it as the “(complex) photocurrent” and presented it with dimensionless normalization^3^ or without normalization^60^ (in the unit mA cm^–2^). Both practices underline the fact that the positive and negative features of *Y*_pc_(*ω*) can be assigned to charge transfer and recombination, respectively, thereby providing a measure for the charge transfer (or injection) or transmission efficiency (see also Section 3.2). This assignment already implies an interpretation of the measurement data, and an assumption that the relation between *J* and *I* is linear (*i.e.*, constant *Y*_pc_(*ω*)) for the whole range of operation parameters. In general, this assumption may not be true since the relation between *J* and *I* is often nonlinear, as will be shown and discussed in detail in Section 4. In order to keep the discussion applicable to any general case and to enable a direct link to *J* as a function of *I* (*J*–*I* curves), we suggest using “*Y*_pc_(*ω*)” (*i.e.*, photocurrent admittance) as the result of IMPS measurements, as presented in eqn (4), without any further interpretation. As specified in ref. 40 (see also ESI,† S3), *Y*_pc_(*ω*) is an admittance. The measured DC value *Y*_pc_(0) indicates the change in *J* due to a change in *I* and represents the slope in the static *J*–*I* curves as illustrated in Fig. 3e. For photoanodes, a large positive value for *Y*_pc_(0) is favored because it signifies a large increase in photocurrent with light intensity.

A typical IMPS spectrum, shown in Fig. 4b, exhibits two semicircles in the positive real half plane of the Nyquist plot, one with a negative imaginary (lower) part and one with a positive (upper) imaginary part. The characteristic frequencies of these semicircles are located where the imaginary part has its extrema. It has been observed that the characteristic frequency of the semicircle in the positive real half plane decreases with increasing series resistance of the PEC cell, as measured by PEIS.^61^ This observation is clarified in detail in the ESI,† S3. The reason why the photocurrent admittance is considered and not the corresponding impedance can be deduced from the typical shape of *Y*_pc_(*ω*). It tends towards zero for *ω* → ∞, which means that the reciprocal function tends to infinity for *ω* → ∞ and therefore the latter does not show a well-defined pattern.

It should be noted that *Y*_pc_(0) is a differential value (see ESI,† S3) and therefore does not give any information on absolute values of the net photocurrent, charge transfer or recombination rates or the corresponding current densities on its own. Instead, it yields the slope of the *J*–*I* curve and also provides information about the characteristic time constants of the related photoelectrochemical processes. In analogy to EIS, it can be stated that absolute numbers can be deduced from one spectrum if, and only if, the system is linear over the whole range. Linearity in this context means that the *J*–*I* curve is a straight line and that *Y*_pc_(*ω*) is constant for a fixed *U* and all light intensities. This is often not the case, as will be shown below.

### 2.4. Intensity modulated photovoltage spectroscopy (IMVS)

As suggested by the name, IMVS probes the relationship between light intensity and photovoltage, at constant current. It is conceptually similar to IMPS (Section 2.3), but it is far less frequently encountered as a measurement technique. As there is no comprehensive introduction on IMVS aimed at photoelectrodes to be found in the literature, the definition we provide here is based on ref. 51 (for DSSC), where a complementary basis of PEIS, IMPS and IMVS was established, which we introduced as photoelectrochemical impedance triplets (PIT) in Section 1.2. As with IMPS, the light intensity, *I*(*t*), is the excitation signal following eqn (2), applied to an IMVS measurement. The current density, *J*, is kept constant, and the measured quantity is *U*(*t*), as illustrated in Fig. 3c. The result of the IMVS measurement is the photovoltage impedance, *Z*_pv_(*ω*):^51^
5
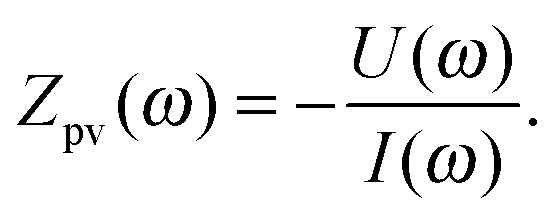

*Z*_pv_(*ω*) is measured in V (W cm^–2^)^–1^, therefore its unit is A^–1^ cm^2^. The DC value at *ω* = 0 is a measure for the relation of potential to light intensity at a fixed photocurrent, *J*, and a small or large *Z*_pv_(0) does not distinguish a high-performing photoanode from a low-performing one, as it is the case for PEIS and IMPS. As can be seen from the iso-photocurrent contour lines in Fig. 3f, *Z*_pv_(0) determines their direction in the surface of operating points introduced in Fig. 2. The negative sign in eqn (5) assures that *Z*_pv_(*ω*) lies in the upper right quadrant in the Nyquist plot, as shown for a typical spectrum in Fig. 4c. If *I* increases, *U* decreases in order to maintain the same *J*. This is in line with the definitions provided in ref. 51 (for DSSC), and the reader is referred to this article and the references therein for a more detailed introduction into IMVS.

To the best of our knowledge, there is currently just one article that discusses IMVS measurements of hematite photoanodes.^48^ The IMVS measurements in ref. 48 were conducted under open circuit conditions, following the common practice in DSSC.^62^ But hematite photoanodes do not operate under open circuit conditions, therefore it is difficult to relate these measurements to the study of hematite photoanodes *in operando* conditions. Thus, the IMVS spectra presented below were measured under bias, *i.e.* in relevant operation conditions for the photoanode.

### 2.5. Photoelectrochemical immittance triplets (PIT)

The PEIS, IMPS and IMVS measurements represent a trio of complementary measurements, which was introduced in Section 1.2 as the photoelectrochemical immittance triplet (PIT). It is noteworthy that two parts of the PIT, measured about the same static operation point (eqn (1)), contain all the necessary information required to calculate the third one (compare eqn (3)–(5)):^51^
6
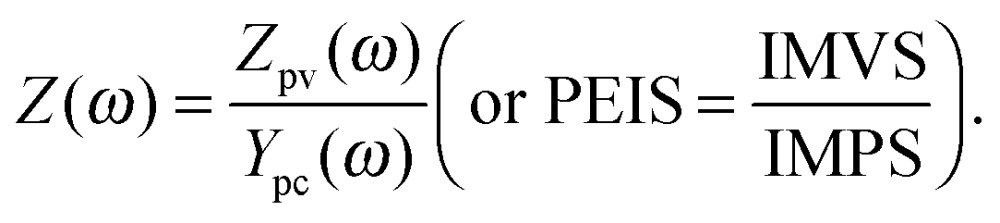
The absence of a minus sign in eqn (6) is counterintuitive but it can be reasoned by considering the gradients in Fig. 3a–c (*Z*(0), *Y*_pc_(0) and *Z*_pv_(0)), which are all positive. Experimental results confirm eqn (6) with good accuracy, as demonstrated in Fig. 4. Thus, it is possible to calculate *Z*(*ω*) from *Z*_pv_(*ω*) and *Y*_pc_(*ω*), calculate *Y*_pc_(*ω*) from *Z*(*ω*) and *Z*_pv_(*ω*), or calculate *Z*_pv_(*ω*) from *Z*(*ω*) and *Y*_pc_(*ω*). In Fig. 4a–c, the measured results are marked by circles, and the “plus” signs indicate calculated results obtained from the respective complimentary measurements (according to eqn (6)). The measured and calculated results overlap each other with good accuracy, except for *Y*_pc_(*ω*) at high frequencies (see Fig. 4b). The deviation originates from the fact that the measured current amplitude *Ĵ* for these data points was very small (<2 μA), because *Y*_pc_(*ω*) tends to zero at high frequency. In fact, the calculated IMPS spectrum in Fig. 4b (“plus” signs) appears to be more sensible because unlike the measured spectrum, it does not reach negative real values. A clearer picture of the quantitative deviation between measured and calculated results is provided by the residual plot in Fig. 4d where the residuals between the measured and calculated results presented in Fig. 4a–c are displayed. The plot shows that the residuals in the region of interest between 0.1 Hz and 100 Hz are small (<2%). Only at frequencies greater than 100 Hz larger residuals are observed, which is attributed to the inaccuracy of the IMPS measurement due to the small current amplitude in this range. That is why we suggest to measure both *Z*(*ω*) and *Z*_pv_(*ω*) under the same operation conditions, and calculate *Y*_pc_(*ω*) from the former two using the relation in eqn (6), in order to obtain the best quality PIT. The validity of this approach is demonstrated in Fig. 4 and also in ref. 51. Kramers–Kronig (KK) residuals calculated for *Z*(*ω*) and *Z*_pv_(*ω*) by the software tool ‘Lin-KK’^63^ are <1% for the whole frequency range (KK residuals and a brief explanation of the KK test^64^ are provided in the ESI,† S3). It is noted that ‘Lin-KK’ does not allow for positive imaginary values in its current version. It is therefore not possible to check the validity of *Y*_pc_(*ω*). However, if it is calculated from the KK compliant measurements of *Z*(*ω*) and *Z*_pv_(*ω*) it must be KK compliant.

## 3. Analysis methods

In this section, we give brief overviews of the well-established techniques for the analysis of PEIS (Equivalent Circuit Model(ing), or ECM, in Section 3.1) and IMPS (the approach developed extensively by Laurence Peter and company,^3,29,37,38,65^ denoted as the Rate Constant Model, or RCM, in Section 3.2), and Distribution of Relaxation Times (DRT, in Section 3.3). In addition to these established analysis methods, in Section 4 we will introduce a DRT-based empirical approach that does not involve any model presumptions, and apply it to analyze our hematite photoanode.

While ECM can be a powerful method for analyzing all three immittance techniques discussed in this article, it is not commonly applied to IMPS and IMVS. Finding the right ECM for a complex system such as a photoanode with complicated morphology and various photoelectrochemical processes involved in the reaction is not trivial, as there is no unique ECM. The fact that an ECM is able to represent a measurement is therefore not a sufficient justification for it to be adopted as a physical model.^66,67^ It is even argued that the electrical circuit viewpoint may not always be the most convenient or physically relevant basis to describe these complex systems, and thus ECMs are merely “analogs” as opposed to being true physical models.^68^ As a case in point, a complex ECM can be fitted to almost every data set^69^ and a very simple model can be used to extract a limited set of parameters from any spectrum. The RCM approach is arguably an improvement in these respects. It begins from an intuitive physical model, and goes a long way in interpreting IMPS data in the framework of this intuitive picture, providing relevant physical or system parameters.^3,6,29,37,38^ Yet it may nevertheless overlook further extent of information or slightly deviating behavior in the corresponding spectrum, as will be demonstrated in Section 3.2.

The third approach, to be introduced in Section 3.3, is empirical, having a minimum of presumptions that can (potentially misleadingly) bias the analysis and interpretation of the results. The measurement results are examined from every possible angle, thereby changing the observer point of view before making any interpretation or assumption. Bode and Nyquist plots are the common techniques to visualize immittance data. The distribution of relaxation times (DRT) is another powerful tool to visualize immittance data, with a high ability to separate polarization processes in the frequency domain, which enables the identification of the different processes. The only *a priori* assumption for the calculation of the DRT is that the immittance measurement is valid, which is equivalent to compliance with the Kramers–Kronig relations^63^ (see also Section 2.5), a general requirement for the validity of immittance data.

**Fig. 5:**
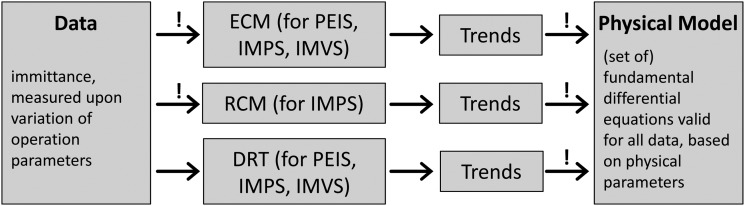
Flow chart of common impedance analysis approaches: ECM (upper branch), rate constant model for IMPS after Laurence Peter (RCM, middle branch), and the here presented empirical approach (DRT, lower branch). All steps in this flow chart that involve presumptions are marked with an exclamation point (“!”). The lower route is presented in this article.

Both the ECM and RCM approaches require the assumption of a model to fit the data as marked by the exclamation point in Fig. 5, where three ways are illustrated how to obtain a model based on physical parameters (physical model) from immittance measurement data. Trends, such as dependencies on operation parameters, cannot be identified without *a priori* assumptions for the ECM and the RCM. Interpreting the parameter trends to deduce a physical model is the final step of all three approaches. It should be emphasized that the first step, deciding on the general analysis approach, is crucial for all further analysis steps and also influences the result. By choosing a rigid model from the outset, any behavior in the data that deviates from it could be ruled out as an error, making it difficult to detect potentially valid system trends.^67^

### 3.1. Equivalent circuit modeling (ECM)

Equivalent circuit modeling (ECM, also: equivalent circuit model) is an important and very popular tool in impedance analysis. It has been applied for hematite photoanodes by several groups. The most recognized work was done by Klahr *et al.*,^7,41,42^ who presented an elaborate model that reproduces the impedance response for a large variety of operating points.^42^ Earlier works by Peter^47^ and Schefold^39^ also apply ECM to PEIS and IMPS for InP photocathodes, with Schefold giving a detailed introduction of how to construct an ECM for both PEIS and IMPS at once.

The concept of ECM is to design a connected network of electrical circuit elements (resistors, capacitors and inductors) that exhibits the same dynamic behavior as the tested system with respect to small signal AC input and output. An ideal electrochemical process is fully characterized by its time constant *τ* and its polarization loss *R*,^40^ and can be modeled by a parallel connection of a resistor and a capacitor. The time constant *τ*, characteristic frequency *f*_c_, resistance *R* and capacitance *C* are related as follows:

7 *τ* = (2π*f*_c_)^–1^ = *R·C*

Since real PEC systems do not always behave as ideal RC circuits, ECMs often include some statistical circuit elements that do not have an exact counterpart in simple electronic circuit elements, such as the constant phase element (CPE).^70^ On the other hand, Gerischer, Warburg or Bisquert elements are simplifications of complex networks that are commonly employed for porous electrodes, for example. More detailed information about ECM can be found elsewhere.^40,54^

**Fig. 6:**
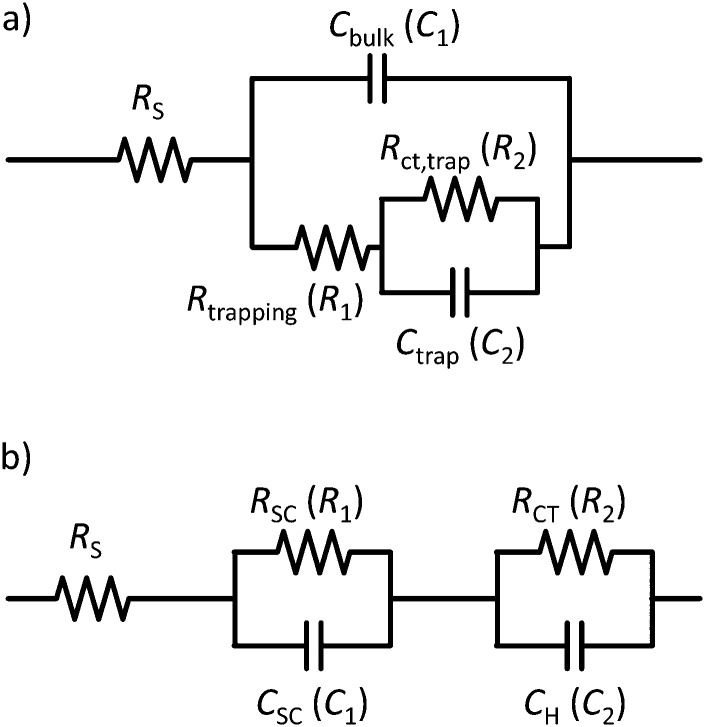
Two examples for common ECMs applied to hematite photoanodes: (a) taken from ref. 42 is the most popular ECM for hematite photoanodes in recent publications; (b) taken from ref. 44. Exemplary fitting results for the spectrum in Fig. 4a are provided in the ESI† (Table S1) using the element names in the brackets.

For the case of PEIS, resistors and capacitors have analogous physical counterparts in the cell, such as the space charge capacitance, Helmholtz capacitance and the charge transfer resistance. However, there is no general agreement on one basic ECM for hematite photoanodes.^42,44,71^ In Fig. 6, the two most recognized ones are displayed, taken from ref. 42 and 44. As demonstrated in ref. 72 these ECMs are in fact equivalent since any spectrum with two time constants can be fitted equally well to both of them. Therefore, a good fitting result for an individual data set fitted to one of the ECMs in Fig. 6 is not sufficient for any conclusion. In general, proposed candidates for ECMs have to be fitted to a series of spectra measured upon variation of at least one operation parameter, in order to extract trends in the behavior of the individual lumped elements in the ECMs. These trends may be able to tell if one model is more sensible than the other one. The extensive parameter study by PEIS in ref. 42 is an adequate example for such a parameter variation. Even so, trends such as the evolution of “*R*_ct,trap_” with potential, cannot be explained satisfactorily, and the nature of this complicated behavior remains unclear. Also, modeling results based on the ECM in Fig. 6a and applying the relations proposed by Peter *et al.* ^6,29,47^ yield surprisingly low charge transfer efficiencies for potentials far beyond the onset potential.^73^

Another issue with the ECMs shown in Fig. 6 is that they feature real capacitors. With those, a satisfactory fit with real measurement data is usually not possible, due to the fact that they exhibit depressed semicircles rather than perfect ones. This only becomes obvious if *Z*(*ω*) is displayed in a Nyquist plot with equally scaled axis, as it is done in Fig. 4a. Better fits are possible if the capacitors in Fig. 6 are replaced CPEs as done in ref. 45 for the model shown in Fig. 6b. This allows for better fits but complicates interpretation.^70^ In the ESI,† S4, it will be demonstrated how the measurement shown in Fig. 4a can be fitted to the ECMs in Fig. 6, with and without CPEs including the impact on the residuals. In general, a meaningful impedance analysis should feature the residuals of the fits. The residuals should exhibit small errors that are randomly distributed. This is explained together with the example in ESI,† S4.

### 3.2. Rate constant model (RCM) for IMPS analysis

Pioneering work in the field of IMPS was done by Peter and co-workers.^3,29,37,38,65^ The most detailed and extensive introduction is given in ref. 29. In addition to describing the measurement technique, the authors introduce a simple model to account for the features in the IMPS spectra. This model, which we will refer to as the RCM herein, has been applied to the major part of IMPS studies on hematite photoanodes to be found in the recent literature.^48,60,73,74^ Ref. 60 is a very good example of the application of IMPS to study hematite photoanodes, providing a comprehensive update on the IMPS theory in a well-accessible and condensed manner. According to the RCM, the charge transfer current density, *j*_t_, and the recombination current density, *j*_r_, are defined as:^29^

8 *j*_t_ = *k*_t_·*Q*_s_.

9 *j*_r_ = *k*_r_·*Q*_s_.

*Q*_s_ is the charge accumulated at the surface of the photoelectrode. The RCM is used to quantify the following parameters:

(1) *k*_t_ (rate constant for charge transfer, see eqn (8)),

(2) *k*_r_ (rate constant for surface recombination, see eqn (9)),

(3) *η*_t_ (charge transfer efficiency, see eqn (10)).

For the identification of the rate constants *k*_t_ and *k*_r_ from an IMPS spectrum, the low frequency intercept with the real axis (LFI), the high frequency intercept with the real axis (HFI) and the value of *ω*_max_ are extracted from *Y*_pc_(*ω*), as indicated in Fig. 4b. A precondition is that the time constants for the two semicircles vary by more than two orders of magnitude.^37^ *ω*_max_ is the angular frequency for which the imaginary part reaches its (positive) maximum. It should be noted that some authors simply use the frequency of the measured point of the spectrum with maximum imaginary part as *ω*_max_. However, if none of the measured points hits the maximum exactly, there can be a significant deviation. The maximum deviation depends on the measured points per decade. The rate constants *k*_t_ and *k*_r_ can be determined with the help of the following two equations:^3,29,37,38,60^
10
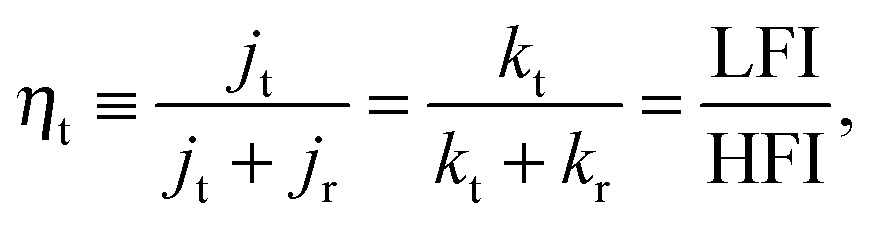

11 *ω*_max_ = *k*_t_ + *k*_r_.

The rate constants *k*_t_ and *k*_r_ are in fact effective (or pseudo) rate constants as they do not describe an individual reaction step but rather the multiple steps of the corresponding chemical reaction, as explained in detail in ref. 47 and 65. There, a theoretical derivation of the individual rate constants for the hydrogen evolution reaction on photocathodes is given. Eqn (8) and (9) further suggest that *k*_t_ and *k*_r_ are invariant rate constants that do not depend on light intensity. This assumption was already proven wrong in ref. 6 and 65. It was shown in ref. 6 for hematite photoanodes, that *k*_t_ and *k*_r_ can change considerably and in a different manner with light intensity. Therefore the rate constants *k*_t_ and *k*_r_ have to be considered as functions of the light intensity, *I* (and as functions of potential as shown in ref. 3, 37, 48, 60, 73 and 74). Also, the equivalences in eqn (10), as suggested in ref. 3, are not fulfilled if *k*_t_ and *k*_r_ are not constant with light intensity. In this case, the charge transfer efficiency *η*_t_ in eqn (10), if determined from an IMPS spectrum measured at a certain *I*_0_, only applies to the additional (*i.e.* incremental) carriers that transfer or recombine in response to a small change in light intensity. Similarly, the current densities in eqn (10) have to be considered as the associated incremental current densities. It should be noted that the measured nonlinearity in the photocurrent with respect to the light intensity is not very pronounced for the case of thin film hematite photoanodes (see Section 4.3). Nevertheless, we consider it one of the reasons that this analysis occasionally yields higher charge transfer efficiencies (eqn (10)) than expected.^73,75^ Therefore, we will suggest another method to determine *η*_t_ in Section 4.3.

As an alternative to IMPS, chopped-light measurements are also proposed to determine the charge transfer efficiency.^60,76^ Through such a measurement, *η*_t_ can be determined as “the ratio of the steady state photocurrent to the instantaneous photocurrent, *j*_ss_/*j*_(*t*=0)_”,^60^ where “*j*_(*t*=0)_” is also described as the “instantaneous hole current”^3,38,60^ and represents the maximum peak current after switching on the light. This method is discussed in detail in ref. 38. It is important to note that the RCM derives *η*_t_ from (small signal) IMPS measurements, whereas the chopped-light measurements usually apply an amplitude of 1 Sun. It is argued in ref. 3 and 60 that the large amplitude can influence the band bending considerably and deteriorate the results and this is why IMPS is favored to determine *η*_t_. However, despite these issues, chopped-light measurements are supposed to yield the global charge transfer efficiency with respect to the chopped-light source, which is usually a solar simulator. In contrast, the RCM yields the charge transfer efficiency with the issues explained above and for the specific light source used for the IMPS measurement (this is further discussed in Section 4.3). The often neglected experimental issues for conducting flawless and reliable chopped-light measurements are beyond the scope of this article.

For the sake of completeness, it should be mentioned that *Y*_pc_(*ω*) is often normalized by *j*_(*t*=0)_ (“*j*_hole_”^37^). Then the low frequency intersect with the real axis represents the transfer efficiency directly, *k*_t_/(*k*_t_ + *k*_r_).^37^ In addition, the ratio of LFI/HFI is often considered equal to “*j*_ss_/*j*_(*t*=0)_”,^3,38,60^ as obtained from chopped-light measurement. The framework introduced by Peter also includes a description for PEIS results,^6,29,47^ which has attracted some interest recently.^73^ However, due to its complexity and rare use it is not discussed any further here.

In conclusion, the RCM is a very useful, elegant and powerful approach to easily extract rate constants for charge transfer and recombination processes and the charge transfer efficiency. However, it assumes a rather simplified model for the kinetics at the photoelectrode/electrolyte interface, which cannot account for nonlinearities in the photocurrent with respect to light intensity or rate constants that depend on light intensity. The RCM serves well for a qualitative comparison of different cells measured under the same operation conditions.^37,48,60,73^ Yet quantitative analyses have to be conducted cautiously: the absolute values for the rate constants and the charge transfer efficiency might not be accurate and potentially vary for different measurement setups or applied analysis methods.

### 3.3. Distribution of relaxation times (DRT) analysis

Several approaches have been used to extract as much information as possible from impedance data.^54^ One of these approaches is the distribution of relaxation times (DRT) analysis, which is a distribution function that can be calculated for any impedance spectrum without any *a priori* assumption.^77^ The most useful characteristic of DRT analysis is its capability to separate polarization processes more clearly than in common Nyquist or Bode plots, where they often appear convoluted. Hence DRT analysis is a powerful tool to support impedance data analysis. It circumvents the construction of ECMs, which always depend on presumptions and are never unique. DRT analysis constitutes the basis of our empirical analysis approach presented herein. Its display can be viewed as “fingerprinting” of the underlying processes and the full potential of the approach is achieved if a series of impedance measurements under different operation conditions are analyzed together. Then each of the individual processes taking place in the photoelectrochemical reaction can be isolated, and their individual behaviors can be followed as a function of the operation conditions.

**Fig. 7:**
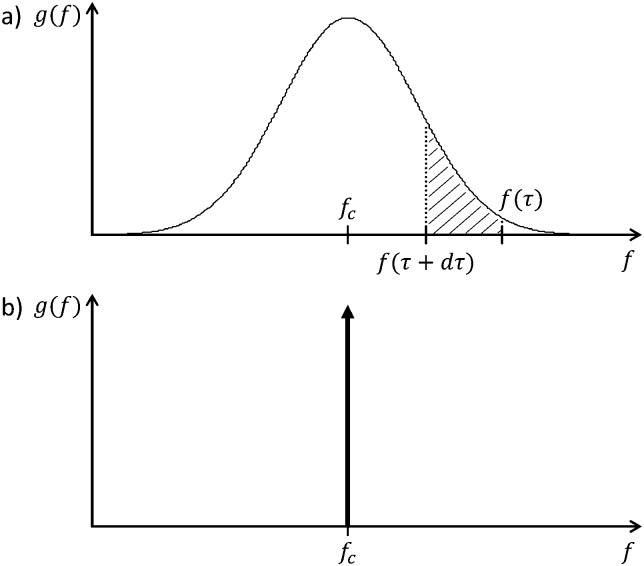
DRT functions of (a) a general polarization process (hatched area is the polarization resistance at relaxation times between *τ* and *τ* + d*τ*), and (b) an ideal RC circuit with the characteristic frequency *f*_c_.

The DRT corresponds to a ‘general’ equivalent circuit, consisting of an infinite number of ‘differential’ RC circuits in series.^56,78^ This is a valid representation of the impedance, as it has been shown that every non-oscillating electrochemical process can be approximated by a series of RC elements.^79^ The measured impedance *Z*(*ω*) can then be expressed by an integral equation containing the DRT, *g*(*τ*):12
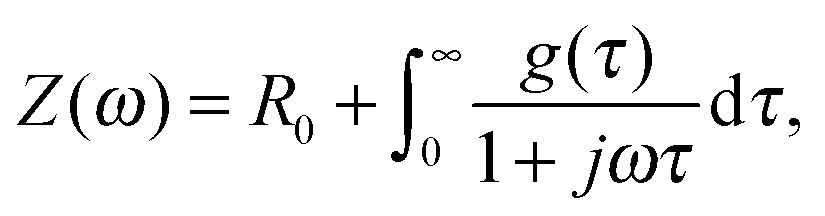
where *R*_0_ is the Ohmic resistance, and the integral represents the total polarization resistance (described in detail in ESI,† S3). For displaying the DRT, *τ* is substituted by the frequency *f* = 1/(2π*τ*), which then leads to a diagram similar to the Bode diagram. The unit for the DRT function *g*(*f*) is Ω s cm^2^ as can be reasoned by eqn (12). The integrand specifies the contribution of any process to the overall polarization at relaxation times between *τ* and *τ* + d*τ*, as shown in Fig. 7a. This implies that the area under each peak in the DRT is equal to the polarization resistance of the corresponding loss mechanism. For a single ideal RC circuit, the DRT shows a Dirac pulse with the area equivalent to its associated resistance, as shown in Fig. 7b.

The calculation of *g*(*τ*) is not a trivial task.^53,80^ It can be performed by Tichonov regularization,^56,81^ which yields good results for the approximation of *Z*(*ω*) in eqn (12). More detailed information about the DRT can be found in ref. 54, 78 and 82.

**Fig. 8:**
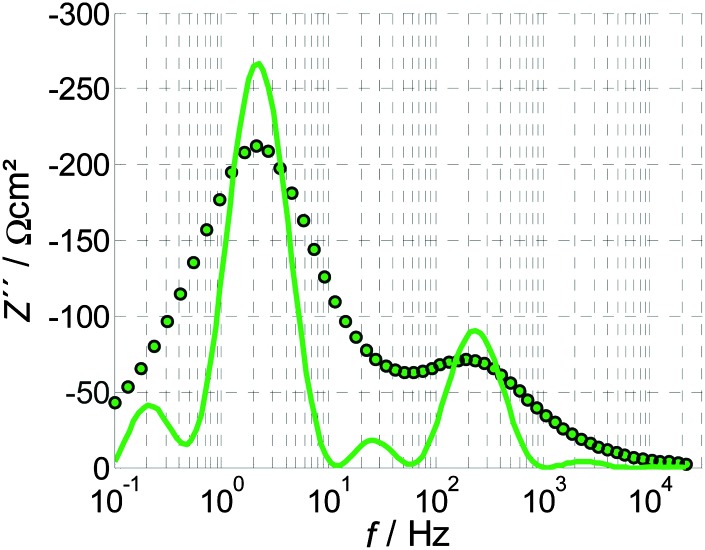
Imaginary part of the PEIS shown in Fig. 4a (circles) compared with the DRT calculated from the same PEIS (line). Note that the DRT function is shown in arbitrary units.

The benefit of the DRT function, *g*(*f*), is demonstrated in Fig. 8, where the negative imaginary part of the impedance spectrum of Fig. 4a is compared to the DRT function calculated from the same spectrum. In this figure, the DRT function exhibits two main polarization processes that can also be identified by the imaginary part of the impedance spectrum at approximately 2 and 200 Hz. However, the DRT function reveals additional smaller peaks at 0.2 and 20 Hz, that correspond to two additional polarization processes. These features are not clearly observed in the imaginary part of the impedance spectrum because they are convoluted with the main peaks. The DRT analysis deconvolves these features, providing essential information for the analysis of impedance spectra without any presumptions as in the previous analysis methods, ECM and RCM. Thus, the DRT analysis is a truly empirical approach.

A lot of important results have been accomplished by the group of Ivers-Tiffée based on the application of DRT analysis for different systems.^54–57,83^ Tsur and co-workers have developed a method to derive a model directly from impedance data with the help of the DRT, by searching for patterns in the DRT function that correspond to a library of impedance elements.^84,85^ The DRT can also be used to assist nonlinear least square fit (CNLS) procedures.^86^ Boukamp has given a recent overview on this field.^87^

Compared to the ECM approach introduced in Section 3.1 and the RCM introduced in Section 3.2, the DRT analysis involves no presumptions, yet it resolves different processes that are often hidden in the immittance data very accurately and comprehensively. As demonstrated in Fig. 8, even small polarization processes are distinguished. However, the DRT analysis is used predominantly for a qualitative assessment of the processes and their dependencies on operation parameters. Absolute quantities are not typically obtained by the DRT analysis itself. Furthermore, the calculation of the DRT function requires advanced mathematical methods and high quality data that is KK compliant with high signal to noise ratio and no drift over time.

## 4. Complementary empirical analysis of PEIS, IMPS and IMVS

In this section, we demonstrate how the information contained in the PITs can be used to gain important insight into the operation mechanism of hematite photoanodes, especially on the forward and backward processes involved in the water photo-oxidation reaction. The experimental procedure can be found in ESI,† S1. Here, we first describe the direct observations by looking at the measurement results in Section 4.1. The DRT analysis is demonstrated on PEIS data in Section 4.2. In Section 4.3, a generalized approach for analyzing IMPS spectra to extract positive and negative current densities is introduced. These will later be shown to correspond to forward charge transfer and backward recombination processes, respectively. We further relate the different techniques gathered in PITs empirically by simple mathematical operations in Section 4.4. Finally, the spectra obtained by these calculations are compared to the measurement and analyzed by DRT in Section 4.5.

All analysis steps are empirical and require only a minimum of basic presumptions. Starting from a simple description, additional techniques and calculation procedures will be developed to complement a detailed framework for empirical analysis of PEIS, IMPS and IMVS. The findings are predominantly geared towards hematite photoanodes, which are used here as a case study, but since no model assumptions are involved, they can be readily adapted to other PEC systems.

### 4.1. Direct observations

**Fig. 9:**
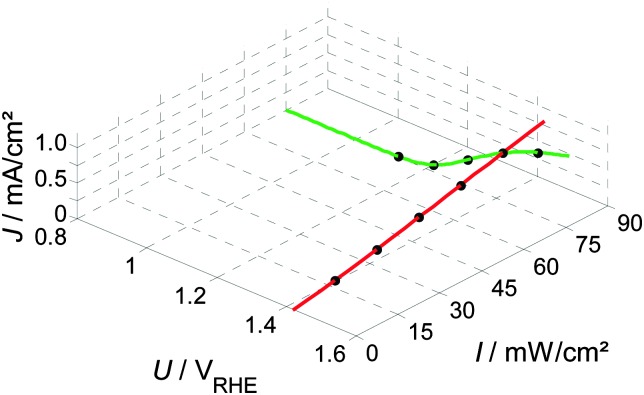
Static photocurrent measurements at constant potential or light intensity (red or green curves, respectively), taken from the 3D map in Fig. 2. Dots indicate, where a PIT was measured.

Fig. 9 shows a reduced version of Fig. 2, with only two curves of constant potential or light intensity (red or green curves, respectively). Those two curves represent the two parameter variations that are discussed here.

**Fig. 10:**
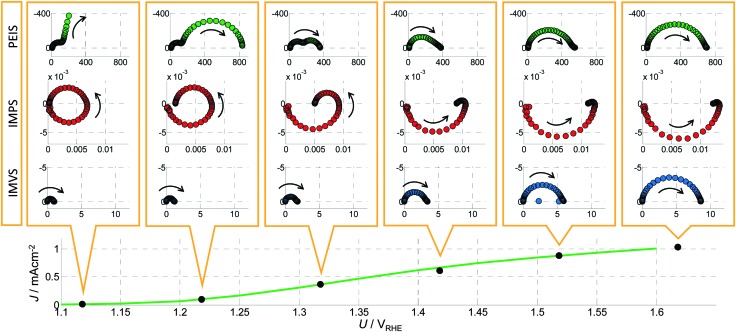
A series of PIT spectra measured at a constant light intensity (*I*_0_ = 75 mW cm^–2^) and different bias potentials (*U*) as indicated by the *J*–*U* curve in the bottom panel (which corresponds to the green curve in Fig. 9). From top to bottom: Nyquist plots of PEIS (green circles, unit: Ω cm^2^), IMPS (red circles, unit V^–1^) and IMVS spectra (blue circles, unit: A^–1^ cm^2^). The arrows point to the direction of decreasing frequency.

**Fig. 11:**
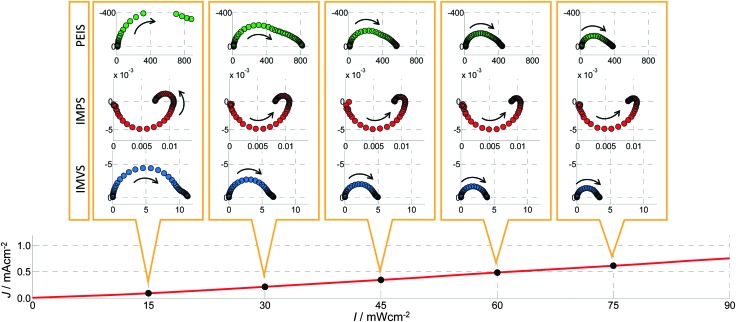
A series of PIT spectra measured at a constant potential (*U*_0_ = 1.42 V_RHE_) and different light intensities (*I*) as indicated by the *J*–*I* curve in the bottom panel (which corresponds to the red curve in Fig. 9). From top to bottom: Nyquist plots of PEIS (green circles, unit: Ω cm^2^), IMPS (red circles, unit V^–1^) and IMVS (blue circles, unit: A^–1^ cm^2^). The arrows point to the direction of decreasing frequency.

Fig. 10 and 11 show PIT spectra at every indicated point along the red and green curves, respectively. These were measured separately from the measurements presented in Fig. 4, therefore there are some small differences between the respective results.

#### PEIS spectra

*Z*(*ω*) shows large DC resistances (*Z*(0)) towards lower potentials (<1.25 V_RHE_) and towards higher potentials (>1.6 V_RHE_) in Fig. 10. This is in accordance with the static measurement shown in Fig. 9, which exhibits a steep increase between 1.25 and 1.6 V_RHE_. At 1.32 V_RHE_, two semicircles are clearly visible. For lower potentials, the semicircle on the right (lower frequencies) seems to dominate, while only one main (approximate) semicircle exists for higher potentials. The latter approaches the real axis at low frequency (*ω* → 0) with an angle significantly smaller than 90°, which is an indicator for a hidden low frequency process. For a change in light intensity *I* at a constant potential of *U*_0_ = 1.42 V_RHE_, shown in Fig. 11, the same shape is visible and the spectrum is just increasing in magnitude towards smaller *I*. Note that the fourth spectrum in Fig. 10 is the same as the fifth spectrum in Fig. 11.

These results are in good agreement with other studies in the literature. For illuminated hematite photoanodes, two well-defined semicircles have been reported and fitted to an ECM, and physical parameters have been extracted.^41^ However, unequivocal assignment of the semicircles to well-defined physico-chemical processes remains a challenge.

#### IMPS spectra

All measured *Y*_pc_(*ω*) spectra in Fig. 10 and 11, as well as in Fig. 4c, are composed of two clearly resolved semicircles. A high frequency semicircle is situated in the lower right quadrant, which we refer to as *Y*_pc_^+^(*ω*), and a low frequency semicircle is found in the upper right quadrant, which we call *Y*_pc_^–^(*ω*). For decreasing frequency, the magnitude of *Y*_pc_(*ω*) increases, then reaches a maximum, before it decreases again to its DC value, *Y*_pc_(0). For a first qualitative description, we regard *Y*_pc_^+^(*ω*) and *Y*_pc_^–^(*ω*) as separate processes and adapt the terminology commonly used for RC circuits§
§The terminology “RC circuit” is not used in a physical sense here. Rather, it is used as a well-established description for the dynamic behavior of a general low-pass filter with a gain *k* and a time constant 
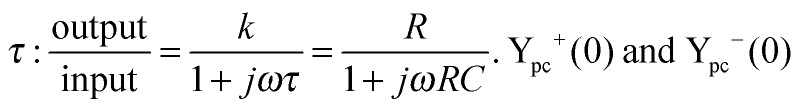
 correspond to *R* or *k* in this equation, but are steady-state admittances in a physical sense. to provide a better understanding. However, we want to emphasize that we omit any presumptions at this point. The diameters of *Y*_pc_^+^(*ω*) and *Y*_pc_^–^(*ω*) are referred to as *Y*_pc_^+^(0) (positive scalar) and *Y*_pc_^–^(0) (negative scalar, considering the direction from high to low frequency), respectively. *Y*_pc_^+^(0) increases only slightly but monotonically from low potentials to high potentials in Fig. 10. *Y*_pc_^–^(0) shows a still monotonic yet more pronounced trend. It is interesting that the abrupt decrease in magnitude of *Y*_pc_^–^(0) coincides with the area of lowest resistance as described above for the direct observation deduced from PEIS measurements. For 1.12 V_RHE_, *Y*_pc_^+^(0) and *Y*_pc_^–^(0) are similar, whereas by 1.62 V_RHE_ *Y*_pc_^–^(0) is greatly diminished. In Fig. 11 it is shown that *Y*_pc_^+^(*ω*) is independent of *I*, whereas the magnitude of *Y*_pc_^–^(*ω*) decreases with increasing *I*.

#### IMVS spectra

The *Z*_pv_(*ω*) spectra look similar in shape for all measurements for the parameter variations in Fig. 10 and 11. All of them exhibit one main semicircle that approaches the real axis at low frequency (*ω* → 0) with an angle significantly smaller than 90°, which resembles the *Z*(*ω*) spectra at high potentials (>1.5 V_RHE_). In Fig. 10 it can be observed that the values for this pattern in *Z*_pv_(*ω*) constantly increase with increasing potential. With increasing *I*, the value of the pattern decreases as can be seen in Fig. 11.

### 4.2. DRT analysis of PEIS

**Fig. 12:**
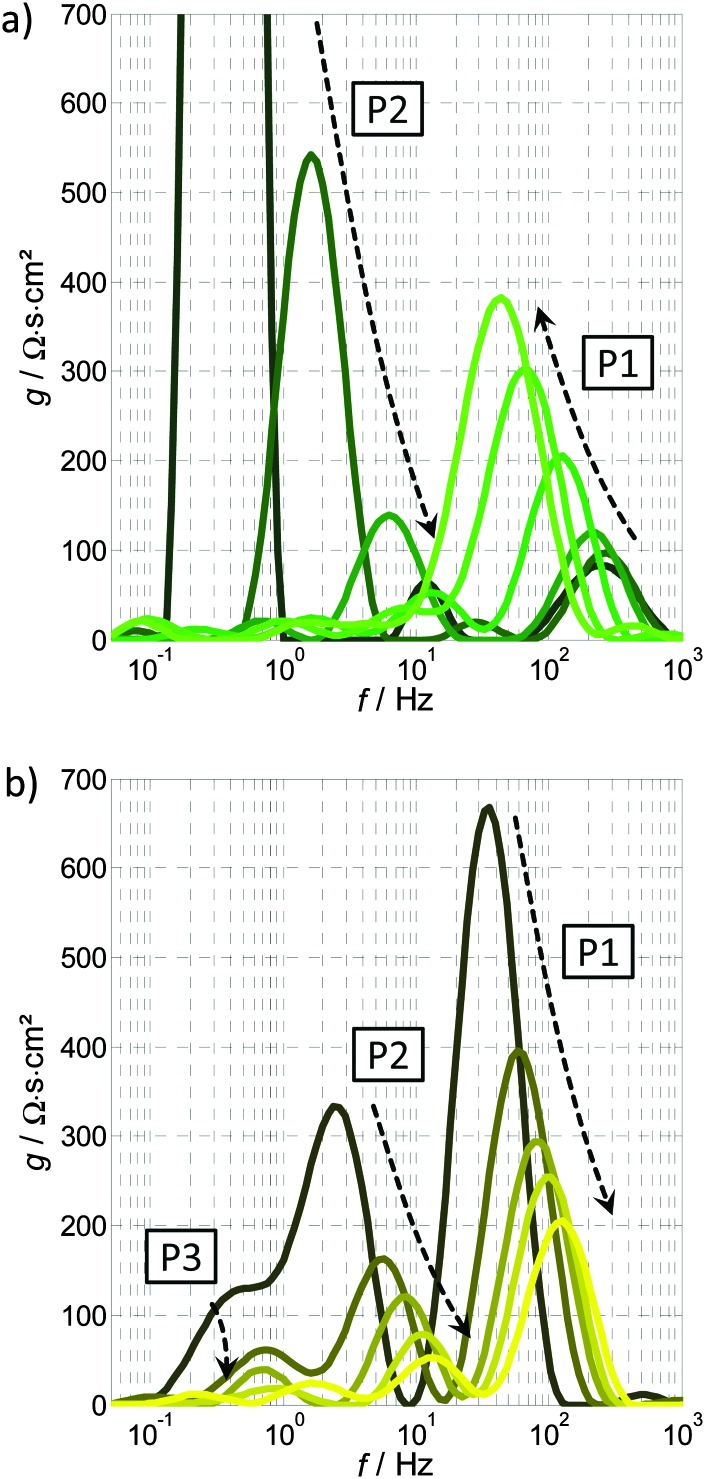
DRT functions calculated from a series of PEIS spectra: (a) variation of the potential, with the arrows indicating an increase in potential from 1.12 to 1.62 V_RHE_ (see also Fig. 10), (b) variation of the light intensity, with the arrows indicating an increase in light intensity from 15 to 75 mW cm^–2^ (see also Fig. 11).

Fig. 12 shows the DRT functions calculated for all the PEIS spectra in Fig. 10 and 11 for the variation of the potential (Fig. 12a, dark green to light green signifies increasing *U*) and light intensity (Fig. 12b, brown to yellow signifies increasing *I*). As already described in Section 3.3, the area underneath each peak is proportional to the resistance of the corresponding process, while the peak frequency is the characteristic frequency of the process. Due to the numerical calculation of the DRT function (*g*(*f*)), there are small oscillations in different frequency regions and the peaks with maximum values of less than 20 Ω s cm^2^ should not be considered.

Both spectra in Fig. 12 show two main peaks that can be related to two governing processes, P1 and P2, which are also responsible for the two distinct semicircles in *Z*(*ω*) in Fig. 10 and 11. Apart from that, there is an additional low frequency process, P3, visible in Fig. 12b. In both diagrams, P1 is clearly distinguishable. It ranges from 30 to 200 Hz and is increasing with higher potential (Fig. 12a) and decreasing with higher light intensity (Fig. 12b), as indicated by the respective arrows. The polarization resistance of process P2 is extremely large for 1.12 V_RHE_ (maximum value 4500 Ω s cm^2^) and almost vanishes for higher potentials, whereas the behavior upon variation of the light intensity is less pronounced (but still clearly visible). The magnitude of P2 decreases with increasing light intensity. P3 in Fig. 12b is decreasing with higher light intensity. The frequency shift of each process can be understood by referring to eqn (7): an increase in *R* causes a decrease in *f*_c_.

The sum of all the polarization processes, *i.e. R*_pol_ = *Z*(0) – *R*_∞_ (see ESI,† S3), is the integrated peak area. *R*_pol_ plus the Ohmic resistance *R*_∞_ correspond, by definition, to the reciprocal of the slope (reciprocal of the derivative) of the *J*–*U* curve in the respective operating point. Based on this fact, the trends in Fig. 12a can be compared with the typical shape of a static *J*–*U* curve: an exponential increase of *J* at the onset potential and saturation at the plateau region towards higher potentials. The behavior of P1 and P2 suggest that P2 is responsible for the photocurrent onset and P1 for the photocurrent plateau in the static *J*–*U* curve. This preliminary result is consistent with findings of Klahr^42^ and, in addition, allows for a clear assignment of the behavior of “*R*_ct,trap_”^42^ (see Fig. 6; mainly responsible for the characteristic shape of the *J*–*U* curve) to two separate processes. “*R*_ct,trap_” decreases with increasing potential below the onset potential and increases with increasing potential beyond the onset potential. These two characteristics seem to be nicely represented by P1 and P2, respectively. Following that, “*R*_ct,trap_” probably contains the lumped resistance associated with P1 and P2 and can be further refined by our DRT analysis.

The general capabilities of the DRT analysis were demonstrated above, as well as the idea behind an empirical assignment of individual processes. We emphasize, however, that at this stage, a physical interpretation is neither desired nor possible solely on the basis of these identification steps.

### 4.3. Extracting positive and negative current densities and the charge transfer efficiency from IMPS spectra

The characteristic shape of the IMPS spectra suggests a straightforward analysis. However, to the best of our knowledge, no other analysis approach for photoanodes than the RCM presented in Section 3.2 exists in the literature. For the analysis approach presented here, we start with a qualitative description of *Y*_pc_(*ω*), to compliment the direct observations in Section 4.1. As already mentioned there, *Y*_pc_(*ω*) exhibits two semicircles in the lower and upper quadrants, denoted as *Y*_pc_^+^(*ω*) and *Y*_pc_^–^(*ω*), respectively. These features are observed in all spectra shown in Fig. 10 and 11. *Y*_pc_^+^(*ω*) has been previously called “attenuation by the PEC”.^3,29,37,60,65^ We do not use this denomination here, as it already involves an interpretation of the results.

The admittance *Y*_pc_(0) is a measure of how much *J* increases with *I*. More exactly, it is, by definition, the slope of the *J*–*I* curve or the derivative of *J* with respect to *I*. Consequently, a positive *Y*_pc_(0) can be related to a positive slope of the *J*–*I* curve, while a negative *Y*_pc_(0) signifies a negative slope in the *J*–*I* curve. With the aim of characterizing individual processes that contribute to the slope, we apply the capability of DRT analysis to separate polarization processes in the IMPS spectra. Toward this end, eqn (12) is modified as follows:13
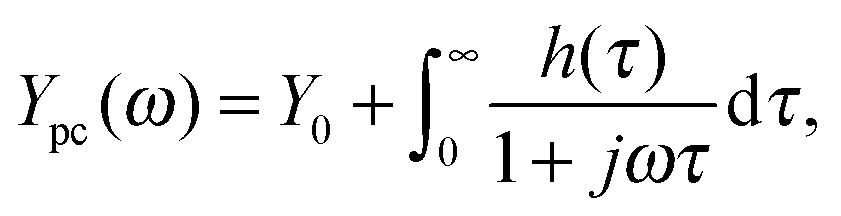
The differences are: *Y*_0_ is negligibly small and the unit of the distribution function *h*(*τ*) is given by V^–1^ s. While eqn (13) shares the same mathematical form as eqn (12), *h*(*τ*) is distinguished from *g*(*τ*). It can be interpreted as the distribution of weighed time constants of the admittance (DTA), indicating with which time constant and to what extent the photocurrent sets in after a step in the light intensity is applied. While we will not delve formally into the concept of DTA here, we proceed to explore its meaning through the example of our model system.

**Fig. 13:**
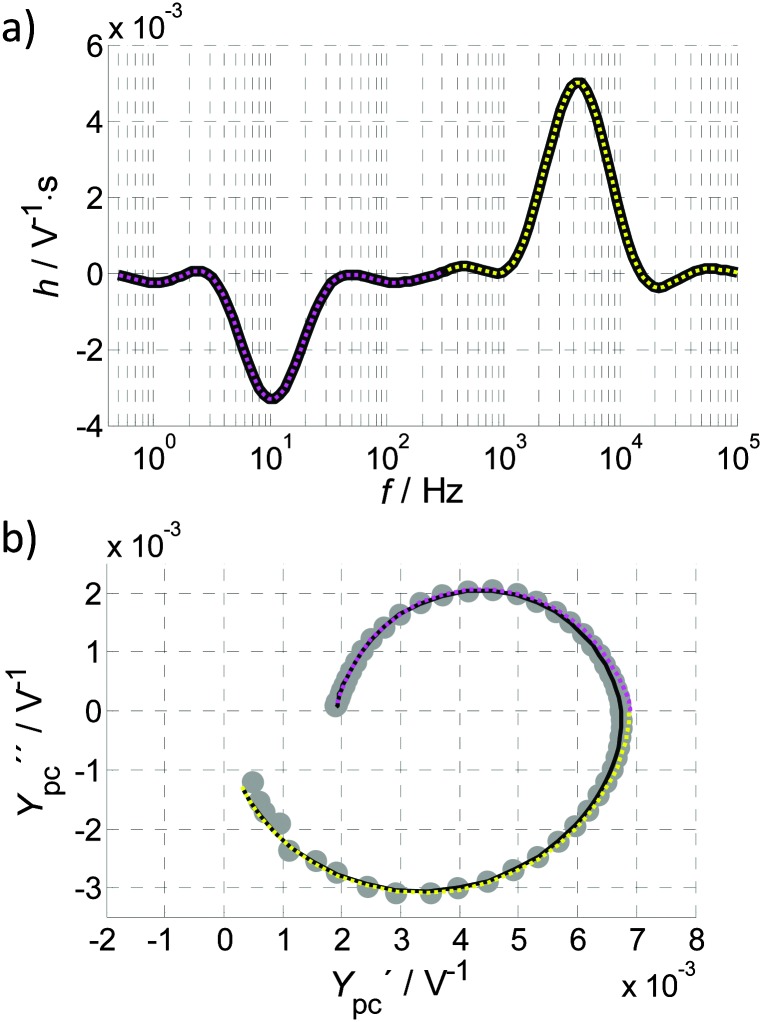
(a) DTA calculated for the IMPS spectrum in Fig. 13b (reproduced from Fig. 4c). The whole DTA function is shown as a black line overlaid by a magenta dotted line that highlights the low frequency part of the DTA (50 mHz to *f*_t_ (300 Hz), while the yellow dotted line marks the high frequent part (*f*_t_ to 100 kHz). (b) IMPS spectrum reproduced from Fig. 4b (grey circles) with the overall fit (black line), obtained from recalculating *Y*_pc_(*ω*) from the DTA function in Fig. 13a by eqn (13) and the corresponding fits from the magenta and yellow parts.

A DTA function calculated from the IMPS spectrum in Fig. 4b, is shown in Fig. 13a, where a positive and a negative process can clearly be distinguished. To separate these processes, a turnover point in the horizontal axis of Fig. 13a, denoted *f*_t_, is chosen (*τ*_t_ = (2π*f*_t_)^–1^). Then the integral in eqn (13) can be divided into two parts:14
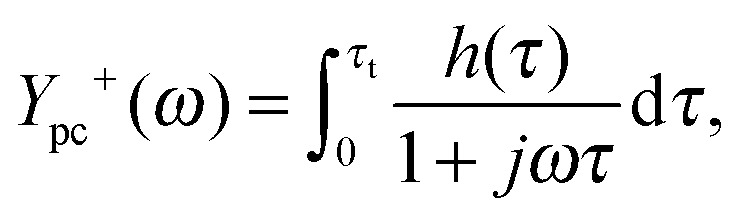

15
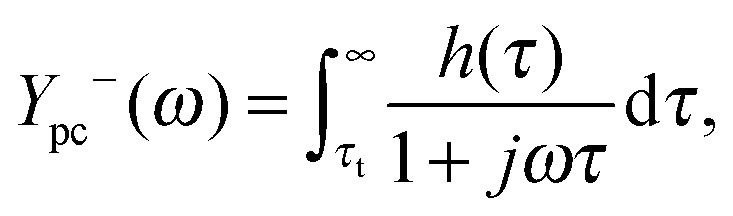
corresponding to the semicircles visible in Fig. 13b. *Y*_pc_^+^(*ω*) and *Y*_pc_^–^(*ω*) as calculated by these equations are plotted independently as the yellow and magenta dotted lines in Fig. 13b, respectively.

For the given example, there are no issues of superimposing peaks in the DTA that might deteriorate the result. This clearly demonstrates that *Y*_pc_^+^(*ω*) and *Y*_pc_^–^(*ω*) do indeed correspond to distinct processes. The black curve in Fig. 13b shows the result for considering the whole DTA function in eqn (13), namely, the complete inverse transformation from the DTA function to the photocurrent admittance. It confirms, by the good agreement with the measurement, that the calculation of the DTA function was accurate and that both the DTA function and *Y*_pc_(*ω*) spectra describe the same dynamic behavior. The two components *Y*_pc_^+^(*ω*) and *Y*_pc_^–^(*ω*) intersect the real axis slightly towards the right, because the frequencies of the two semicircles superimpose in the complete back calculation. For this reason, a normalization of *Y*_pc_(*ω*), as described in ref. 3, 29, 37, 38 and 65 needs to be conducted with care.

One may be tempted to apply a simplified approach to identify *Y*_pc_^+^(0) and *Y*_pc_^–^(0) directly from the IMPS spectrum: considering the intersection to the right with the real axis in Fig. 13b as *Y*_pc_^+^(0) and the difference between this intersect and *Y*_pc_(0) as *Y*_pc_^–^(0). However, the description above shows that only by considering the two contributions *Y*_pc_^+^(*ω*) and *Y*_pc_^–^(*ω*) independently, exact numerical values for the positive and negative contributions of *Y*_pc_(*ω*) can be deduced. The simplified approach only produces a small error in Fig. 13b, but yet it is not exact, and in some cases the error may become significant.

**Fig. 14:**
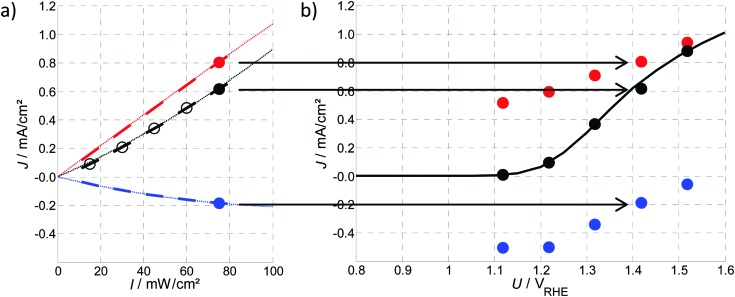
(a) Dotted lines: fitted *J*–*I* curves for 1.42 V_RHE_ (black: *J*(*I*), red: *J* ^+^(*I*), blue: *J* ^–^(*I*)); black circles: measured static values for *J*(*I*); thick lines: local slopes of *Y*_pc_(0) (black), *Y*_pc_^+^(0) (red) and *Y*_pc_^–^(0) (blue); solid circles: fitted values for *J*(75 mW cm^–2^) (black), *J* ^+^(75 mW cm^–2^) (red) and *J* ^–^(75 mW cm^–2^) (blue). (b) Black line: measured *J*–*U* curve for 75 mW cm^–2^; black circles: static values measured during recording PIT spectra); other circles: fitted values for *J* ^+^(75 mW cm^–2^) (red) and *J* ^–^(75 mW cm^–2^) (blue).

As discussed in Section 2.3, *Y*_pc_(0) is the slope of the *J*–*I* curve. Neglecting *Y*_0_ in equation then leads to16
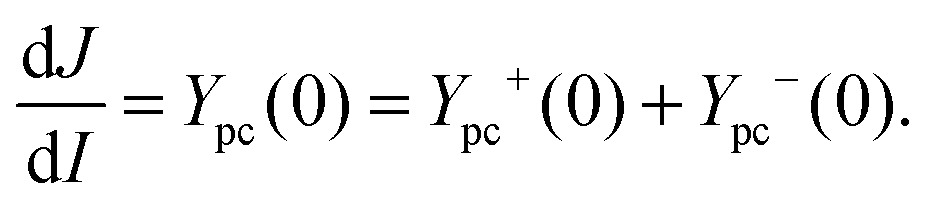
Eqn (16) suggests positive and negative contributions to the photocurrent, which expressed as a function of *I* are denoted *J* ^+^(*I*) and *J* ^–^(*I*). *Y*_pc_^+^(0) and *Y*_pc_^–^(0) thus correspond to the local slopes of *J* ^+^(*I*) and *J* ^–^(*I*), respectively. This accounts for the corresponding operating point, determined by *I* and *U* (see eqn (1)). It then becomes possible to reconstruct the functions *J* ^+^(*I*) and *J* ^–^(*I*) for a given *U*. For this, we extract *Y*_pc_^+^(0) and *Y*_pc_^–^(0) from all the IMPS spectra in Fig. 11 and conduct a linear curve fit for *J* ^+^(*I*) and *J* ^–^(*I*). The available information for this fit is:

• the absolute values of *J*(*I*) (static values obtained from analysis of PIT spectra, indicated by black circles in Fig. 14),

• the relation *J*(*I*) = *J* ^+^(*I*) + *J* ^–^(*I*),

• *Y*_pc_(0) is the local slope of *J*(*I*),

• *Y*_pc_^+^(0) is the local slope of *J* ^+^(*I*),

• *Y*_pc_^–^(0) is the local slope of *J* ^–^(*I*),

• *J*(0) = *J* ^+^(0) = *J* ^–^(0) = 0 (any *J* ≠ 0 can be attributed to the dark current and has to be subtracted from the absolute values of *J*(*I*) prior to this analysis).

All of this information is passed to the linear curve fitting algorithm described in the ESI,† S5 and the functions *J*(*I*), *J* ^+^(*I*) and *J* ^–^(*I*) are obtained. A slightly nonlinear behavior of the *J*–*I* curve is expected because *Y*_pc_(*ω*) differs for a variation in *I*, as can be seen in Fig. 11. The variation of *Y*_pc_(0) in Fig. 11 is around 20% (7.4 × 10^–3^ V^–1^ for 15 mW cm^–2^ and 9.0 × 10^–3^ V^–1^ for 75 mW cm^–2^). A precise analysis of static and dynamic measurements must therefore consider the relation between *J* and *I* as nonlinear. This means that the polynomial has to be of 2nd or higher order. For the given example, an order of 2 yields good results in Fig. 14a, where *J*(*I*), *J* ^+^(*I*) and *J* ^–^(*I*) obtained from the IMPS spectra in Fig. 11 are shown.

**Fig. 15:**
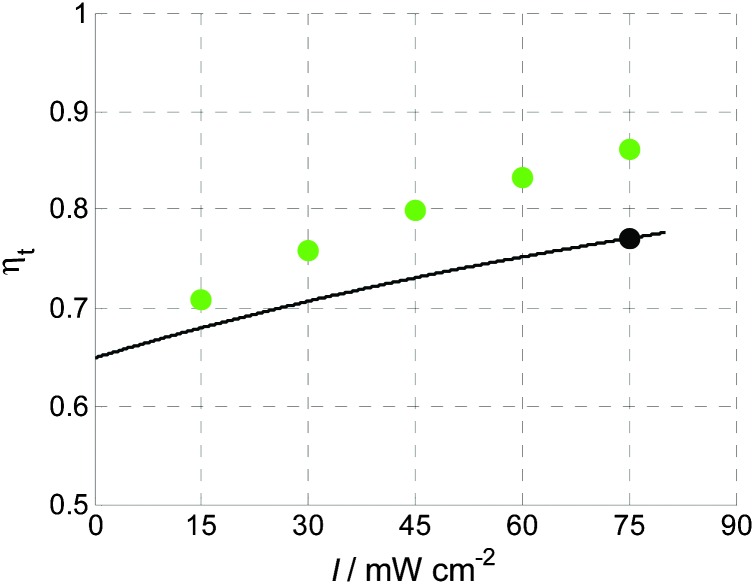
Charge transfer efficiencies, *η*_t_, for 1.42 V_RHE_ calculated by the RCM (green circles) and the empirical model proposed in eqn (17) (straight line). The operating point for the *J*–*U* curve in Fig. 14a at a light intensity of 75 mW cm^–2^ is marked by an extra black circle.

In Fig. 14a the red dotted line, *J* ^+^(*I*), is rather straight, as expected. The blue dotted line, *J* ^–^(*I*), exhibits a certain decay for small *I* and seems to saturate for larger *I*. The errors for this fit appear as deviating directions of the dotted lines and the thick bars (error in the slopes) and the deviation of the black dotted line and the black circles (error in absolute values for *J*(*I*)). As can be noticed, the errors are very small for this example. The values for *J*(75 mW cm^–2^), *J* ^+^(75 mW cm^–2^) and *J* ^–^(75 mW cm^–2^) in Fig. 14a correspond to the point at 1.42 V_RHE_ in Fig. 14b, where *J* ^+^(75 mW cm^–2^) and *J* ^–^(75 mW cm^–2^) were also determined for four other potentials (applying the slightly simplified approach introduced at the bottom of this section). Given that *J* ^+^(*I*) and *J* ^–^(*I*) are defined as the positive and negative contributions, respectively, to the total current *J*(*I*), it is natural to define an efficiency:17
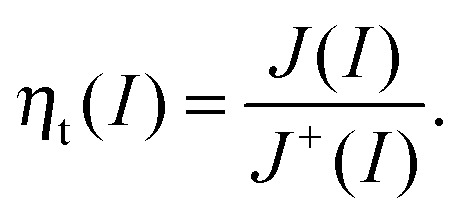
Physically, *J* ^+^(*I*) may be interpreted as the flux of holes that reach the surface, and *η*_t_ as the transfer efficiency (see Section 5), although this has no bearing on the empirical analysis itself. On the basis of the IMPS spectra of Fig. 11, Fig. 15 compares *η*_t_ calculated by eqn (17) with *η*_t_ obtained from the RCM (eqn (8)–(10)).

The values differ between the two methods. This is not surprising considering that eqn (10) cannot be expected to calculate global transfer efficiencies, as discussed in Section 3.2. Nevertheless, the average of the RCM values for *η*_t_ is in somewhat reasonable agreement with the DTA-based *η*_t_. With the DTA approach, *η*_t_ is calculated by the integration of IMPS data, the *J*–*I* curve itself, and yields a value that is 10% lower (at *I* = 75 mW cm^–2^) than the RCM method. Since *η*_t_ changes with light intensity for both methods, it will also be sensitive to the makeup of the light spectrum of the source used for the light bias. This has serious consequences that have been overlooked before. Usually *η*_t_ is determined using the RCM with IMPS spectra measured with a special light source that does not emulate typical operation, as already mentioned in Section 3.2. Thus, the obtained *η*_t_ is not necessarily equivalent to operation under 1 Sun. It should be noted that in our measurements we used a blue LED (*λ* = 449 nm) instead of a spectrum solar simulator, so the results cannot be compared directly to the conditions at 1 Sun. However, the *J*–*U* curve shown in Fig. 14b has been measured with the same LED and therefore the measurement results obtained by all methods shown here have been obtained under comparable conditions.

The fit procedure introduced above can be simplified when considering that *Y*_pc_^+^(0) is almost constant for all *I* in a first approximation (less than 5% variation from 10.5 × 10^–3^ V^–1^ at 75 mW cm^–2^ to 10.9 × 10^–3^ V^–1^ for 15 mW cm^–2^). Consequently, *J* ^+^(*I*) is a line though the origin and only the average of *Y*_pc_^+^(0) for different light intensities is required to find *J* ^+^(*I*). With *J* ^–^(*I*) = *J*(*I*) – *J* ^+^(*I*), also *J* ^–^(*I*) can be obtained. Note that this is not a generally valid simplification as *Y*_pc_^+^(*ω*) is not independent of *I per se*. Different samples or different potentials might also cause a nonlinearity in the function *J* ^+^(*I*). However, when the linearity of *J* ^+^(*I*) is validated, this simplification can reduce the measurement effort enormously, if *J* ^+^ and *J* ^–^ are only required for one *I*_0_, such as in the example in Fig. 14b for the *J*–*U* curve at 75 mW cm^–2^: *Y*_pc_^+^(0) for *Y*_pc_(*ω*) extracted from the IMPS spectrum at *I*_0_ alone determines the linear function *J* ^+^(*I*). From *J* ^–^(*I*_0_) = *J*(*I*_0_) – *J* ^+^(*I*_0_), all three points can be calculated. This strategy was applied for the remaining points shown in Fig. 14b.

A possible interpretation of the findings in this section will be given in Section 5.

### 4.4. Relating IMPS to PEIS and IMVS

As described in Section 4.1, there are several distinct characteristics that can be found in the PIT spectra in Fig. 10 and 11. However, the question remains: how are they related?

**Fig. 16:**
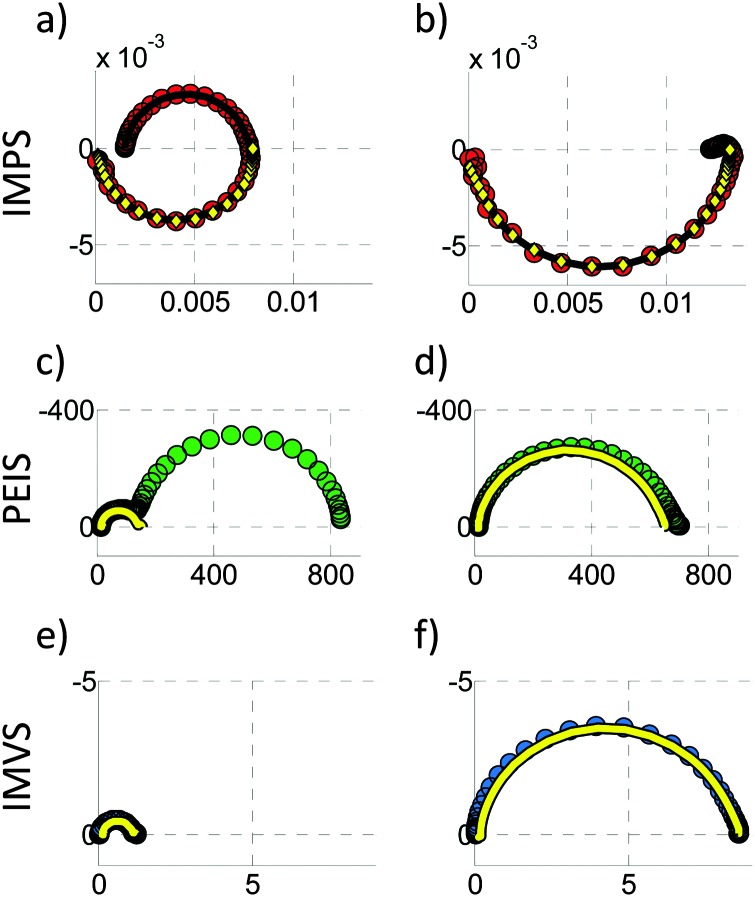
Left side (a, c and e): Spectra recorded at 1.22 V_RHE_ and 75 mW cm^–2^; right side (b, d and f): spectra recorded at 1.62 V_RHE_ at 75 mW cm^–2^. (a and b) *Y*_pc_(*ω*) (red circles) with DTA reconstruction (black line) and *Y*_pc_^+^(*ω*) (yellow diamonds), (c and d) corresponding *Z*(*ω*) (green circles) and *Z* ^+^(*ω*) (yellow line), (e and f) corresponding *Z*_pv_(*ω*) normalized by *Y*_pc_^+^(0) (blue circles) and *Z* ^+^(*ω*) (yellow line).

For this we separate *Y*_pc_^+^(*ω*) and *Y*_pc_^–^(*ω*) again, as described in Fig. 13b. As introduced in eqn (6), it is possible to calculate *Z*(*ω*) from *Y*_pc_(*ω*) and *Z*_pv_(*ω*). In order to elucidate the relationship between *Y*_pc_(*ω*) and *Z*(*ω*), we modify eqn (6) by using only the *Y*_pc_^+^(*ω*) component shown in Fig. 16a and b, in place of *Y*_pc_(*ω*) and define *Z* ^+^(*ω*) as the component of *Z*(*ω*), which is directly related to *Y*_pc_^+^(*ω*):18
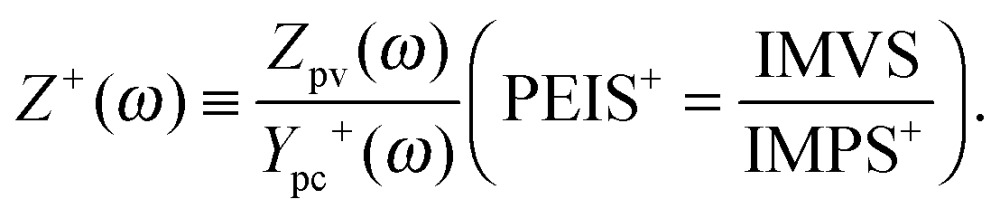
The result is shown as a yellow line in Fig. 16c and d. At 1.12 V_RHE_ (Fig. 16c), it is clear that the (small) high frequency semicircle in *Z*(*ω*) is associated with *Y*_pc_^+^(*ω*) and the (large) low frequency semicircle must be related to *Y*_pc_^–^(*ω*), because the latter disappears when *Y*_pc_^–^(*ω*) is omitted from the impedance calculation of eqn (16). The low frequency semicircle is very small at 1.62 V_RHE_ (Fig. 16d) and consequently, *Z*(*ω*) and *Z* ^+^(*ω*) show a similar shape apart from a small deviation at low frequency.

It was already mentioned in Section 4.1 that the shapes of *Z*(*ω*) and *Z*_pv_(*ω*) are similar for high potentials. To follow up on this observation, we also compare *Z* ^+^(*ω*) and *Z*_pv_(*ω*), normalizing the latter by *Y*_pc_^+^(0) as shown in Fig. 16e and f.

The aforementioned similarity is striking for both potentials. This will be investigated in more detail by a DRT analysis of *Z*(*ω*), *Z* ^+^(*ω*) and *Z*_pv_(*ω*) in Section 4.5, and further discussed in Section 5.

### 4.5. Analyzing PEIS, PEIS^+^ and IMVS by DRT

**Fig. 17:**
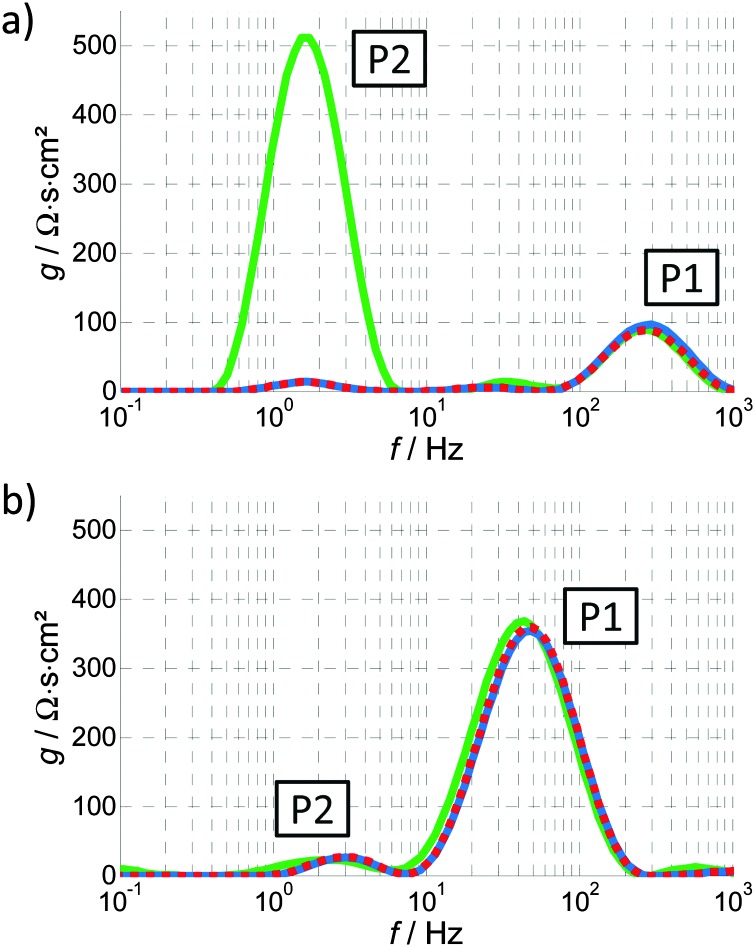
DRT functions for (a) 1.22 V_RHE_ and (b) 1.62 V_RHE_; calculated from *Z*(*ω*) (green line), *Z* ^+^(*ω*) (red dotted line) and *Z*_pv_(*ω*) (blue line, for better comparison, *Z*_pv_(*ω*) is divided by *Y*_pc_^+^(0)).

The similarity of *Z*(*ω*), *Z* ^+^(*ω*) and *Z*_pv_(*ω*) demonstrated in Fig. 16 motivates a more detailed analysis of these three spectra. This is done *via* DRT analysis of these spectra as shown in Fig. 17. The application of eqn (12) to *Z*_pv_(*ω*) is straightforward, since it is technically an impedance after the above mentioned normalization by *Y*_pc_^+^(0).

As introduced in Section 3.3, each peak in the DRT spectra can be attributed to a process whose characteristic frequency and related resistance are given by the peak frequency and area, respectively. Comparing the DRT functions of *Z* ^+^(*ω*) (red dotted line) and *Z*(*ω*) (green line), the following observations are made: for our case study, peak P1 (as indicated in Fig. 17) at 300 and 50 Hz is visible in all of the DRT functions for *U* = 1.22 and 1.62 V_RHE_, respectively. The fact that these can be attributed to the same process is suggested by the smooth transition shown in Fig. 12a, which showed the evolution of the DRT functions of PEIS spectra for the same potential variation. At high potential (Fig. 17b), the pattern for *Z*(*ω*) is also very similar to the pattern for *Z* ^+^(*ω*). At low potentials (Fig. 17a), the situation is different. Here, the similarity of the pattern of *Z*(*ω*) and *Z* ^+^(*ω*) is not valid for low frequencies. A large 2 Hz peak (P2) exists only for the DRT function obtained from *Z*(*ω*). The corresponding Nyquist plot, shown in Fig. 16c (PEIS spectrum at 1.22 V_RHE_), consists of two semicircles and the large peak in the DRT function is related to the large low frequency semicircle. This P2 is almost invisible for high potentials and the corresponding Nyquist plot only shows one distinct semicircle (Fig. 16d). This suggests that P2 is mathematically related to *Y*_pc_^–^(*ω*), since it was omitted for the calculation of *Z* ^+^(*ω*) and it was large for 1.22 V_RHE_ and almost negligibly small for 1.62 V_RHE_.

By comparing *Z* ^+^(*ω*) (red dotted line) and *Z*_pv_(*ω*) (normalized, blue line) in Fig. 17, the following observations are made: the DRT functions of *Z* ^+^(*ω*) and the normalized *Z*_pv_(*ω*) show almost exactly the same pattern. Considering the calculation of *Z* ^+^(*ω*) (eqn (18) as compared to eqn (6)), it means that there is no visible difference in the obtained spectrum, whether *Z*_pv_(*ω*) is divided by *Y*_pc_^+^(*ω*) or *Y*_pc_^+^(0). Next we observe that a comparison of *Z*(*ω*) (green line) and *Z*_pv_(*ω*) (normalized, blue line) also confirms the observation in Section 4.1 (Fig. 10), that the shapes of *Z*(*ω*) and *Z*_pv_(*ω*) assimilate for high potentials. Finally, we observe that only when *Y*_pc_^–^(*ω*) is pronounced in the IMPS plot, P2 in *Z*(*ω*) is present as a peak in the DRT function and as a semicircle in the Nyquist plot.

By this empirical approach – comparing the DRT functions of *Z*(*ω*), *Z* ^+^(*ω*) and *Z*_pv_(*ω*) – it was possible to relate the low frequency semicircle in *Z*(*ω*) to *Y*_pc_^–^(*ω*) mathematically. Also, we have presented both a calculation (*Z* ^+^(*ω*) *via*eqn (18)) and a measurement technique (IMVS, *Z*_pv_(*ω*)) for probing the remaining processes occurring during water oxidation on hematite photoanodes. As eqn (6) and (18) are also valid for the DC case (*ω* → 0), the resistances related to these processes can be calculated easily with these equations. Armed with these observations, a physical interpretation will be discussed in Section 5.

## 5. Discussion

The guiding principle of our empirical analysis approach, as demonstrated in Section 4, has been to organize the PITs in a coherent way, while largely avoiding the need to impose the constraints of a pre-defined model and delaying it to the final step. Indeed, the low frequency P2 peak was only assigned to a polarization process after the empirical analysis provided the clear evidence for it. In Section 4.3 we have also presented a way to separate the positive and negative contributions to *Y*_pc_(*ω*) without any physical interpretation, but showed that they correspond to positive and negative processes in the DTA spectrum. If we now consider the corresponding functions *J* ^+^(*I*) to be the current density of holes that reach the surface and *J* ^–^(*I*) as surface recombination current,^3,29,37,65^ this procedure gives access to these elusive but physically relevant quantities which describe the operation mechanism of hematite photoanodes. The approach in Section 4.3 introduced IMPS and the related analysis as handy tools to get access to the negative recombination current as a function of the operation conditions, *in operando*, and with no need to use sacrificial reagents (*e.g.*, H_2_O_2_) that give rise to parasitic side effects and reactions. As the measurements and fits are of excellent quality, the negative recombination current can be determined with very good accuracy.

With the analysis of Sections 4.4 and 4.5, direct correspondence was demonstrated between *Y*_pc_^–^(*ω*) and the P2 peak in the DRT function of *Z*(*ω*). These considerations strongly suggest that the P2 peak in both Fig. 12 and 17 is related to surface recombination. It follows that the low frequency semicircle of *Z*(*ω*) is caused by surface recombination. We have therefore found a credible way to assign this feature in the PEIS spectrum to a physical process, without imposing model presumptions at the beginning of the analysis, which may give rise to false conclusions. This result amounts to an empirical identification of the low frequency process.

**Fig. 18:**
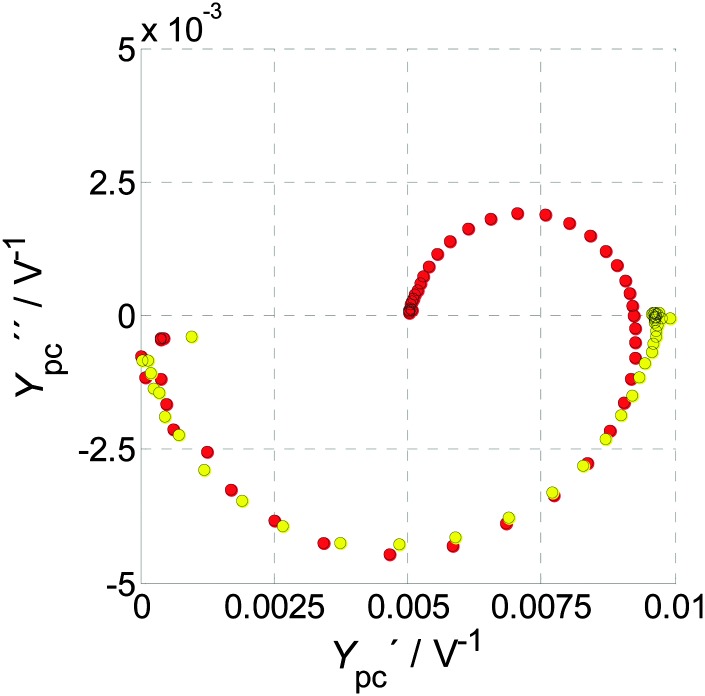
IMPS measurements in 1 M NaOH at 1.32 V_RHE_ (red circles) and in 0.5 M H_2_O_2_ + 1 M NaOH at 1.02 V_RHE_ (yellow circles). Both measurements were conducted at 75 mW cm^–2^.

Our empirical analysis approach enables us to identify surface recombination in both PEIS and IMPS spectra with the only assumption being that the negative component in IMPS is related to this process, which is also suggested by the RCM.^3,29,37,47,65^ Further support for this interpretation is provided by Fig. 18, where IMPS measurements with and without H_2_O_2_ as hole scavenger^8^ are presented. The measurement without H_2_O_2_ clearly shows a negative semicircle (*Y*_pc_^–^(*ω*), see Section 4.1), whereas the one with H_2_O_2_ does not. Similar results can also be found elsewhere.^48,74^ The magnitude of the positive semicircle (*Y*_pc_^+^(*ω*)) is almost equal for both measurements, which lets us conclude that the only difference between the measurements is inactivated surface recombination for the measurement with H_2_O_2_. The different potentials applied during the measurement correspond to changes in the surface potential due to surface charging, which will be discussed in detail in a future publication.

Furthermore, we have demonstrated in Sections 4.4 and 4.5 that IMVS spectra exhibit similar patterns as PEIS spectra. At higher potentials, where the effect of surface recombination in PEIS is negligible, the characteristics of PEIS and IMVS vary only by *Y*_pc_^+^(0). This suggests that IMVS can be seen as being proportional to PEIS, minus the effect of surface recombination.

The consequences for the analysis of PIT (PEIS/IMPS/IMVS) are remarkable. We now have an additional tool at our disposal, since we can measure IMVS and obtain PEIS, but without the low frequency recombination process. To appreciate this newfound benefit, we compare it to the fit strategy proposed by Boukamp:^88^ the latter approach is to identify single processes in the impedance spectrum, and successively subtract them in order to get a better accuracy for the remaining processes. By measuring IMVS instead of PEIS, the same outcome can be achieved through direct measurement, but without uncertainties associated with the process identification and subtraction procedure. The trends of the remaining processes can then be analyzed with a much better accuracy and confidence, as crosstalk from surface recombination is eliminated.

The result that IMVS is equal to PEIS after subtracting the impact of surface recombination might be puzzling in the first place, considering that IMVS is used to directly probe the dynamics of charge carrier recombination in DSSC.^51,62^ However, these measurements are conducted in open circuit without current, so that the charge carriers have no path to go, other than to recombine with each other. The resultant behavior cannot be compared with photoanodes *in operando* as studied here, which are operated under bias that gives rise to a finite photocurrent.

To understand the similarities in the PEIS and IMVS spectra in a simple way, consider the dynamic relations in both techniques. PEIS measures (for small signals) the ratio of voltage to current, while IMVS measures the ratio of voltage to light intensity. Therefore, if we were to assume that the current density of holes that reach the surface is proportional to light intensity (for small signal modulation), it follows that the denominators of both ratios (eqn (3) and (5)) should be proportional, and hence IMVS should be proportional to PEIS. However, this only holds true, if the influence of the recombination current, which is not a direct proportionate of the light intensity, can be neglected. This is the case for high bias potentials. With this modified picture, the observed result becomes plausible, that IMVS should be proportional to PEIS, but without the contribution of recombination.

## 6. Conclusions

Based on three complementary photoelectrochemical immittance techniques – PEIS, IMPS and IMVS, denoted as photoelectrochemical immittance triplets (PIT) – an empirical analysis of the polarization process underlying the operation mechanism of hematite photoanodes is possible. It avoids ambiguities inherent in other approaches, which are commonly used in the recent literature.

PIT comprises the response to a variation of the most important operation parameters: potential and light intensity. PIT measurements can be done under the exact same conditions as the operation conditions of the photoelectrode, and are therefore well-suited to analyze its dynamic behavior *in operando* without altering anything in the system. They are able to probe elusive quantities such as the hole current density and the negative surface recombination current density. We interpret the obtained results such that we have found a way to separate the influence of surface recombination unequivocally from all other losses in the impedance spectrum. IMPS was presented as a suitable technique to quantify positive and negative contributions to the photocurrent. On the basis of this framework, a deeper understanding of the forward and backward processes from PEIS measurements is provided.

Additionally, we have shown that IMVS is a suitable, yet under-utilized, technique for probing the behavior of hematite photoanodes. The measurement is easy to conduct and yields a very clear result. It was illustrated that the IMVS spectrum contains equivalent information as the PEIS spectrum without the influence of surface recombination. Thus, it alleviates the need to fit PEIS data to complex ECMs, identify surface recombination and search for a suitable model for the remaining processes. All remaining processes can simply be measured directly by IMVS. This constitutes a great opportunity for further analyzing the processes that limit the photocurrent at potentials beyond the onset potential, which is necessary for a better understanding of the oxygen evolution reaction on hematite photoanodes.

The results from the analysis presented herein are in line with recent literature. Beyond that, our empirical approach avoids pressing measurement results into standard schemes of ECM and is even more flexible than the rate constant model (RCM) for IMPS analysis developed by Laurence Peter. Therefore, our approach is very general and can potentially be applied to many other PEC systems as well.
