# Supplementary material for: Empirical in operando analysis of the charge carrier dynamics in hematite photoanodes by PEIS, IMPS and IMVS
Source: Phys Chem Chem Phys. 2016 Aug 1;18(34):23438–57. doi: 10.1039/c6cp04683e (PMC5310524; doi:10.1039/c6cp04683e)
Supplement: Supplementary file 1 [file CP-018-C6CP04683E-s001.pdf]

Electronic Supplementary Material (ESI) for Physical Chemistry Chemical Physics.
This journal is © the Owner Societies 2016

Supporting Information

S1. Measured Samples and Test Setup

All the measurements reported in this work were carried out with an undoped hematite photoanode that was deposited by spray pyrolysis on fluorine doped tin oxide (FTO) coated glass substrate (TEC 15, Hartford Glass). The sprayed solution contained 10 mM of $Fe(acac)_3$ (99.9%, Aldrich) in EtOH (99.8% Fluka). On the first stage, a bottom layer was deposited. During the bottom layers' deposition, two columns of three substrates (six in total) were subjected to 5 ml of solution spray. For the top layer deposition, a new $Fe(acac)_3$ in EtOH solution was prepared. During the top layer deposition, two columns of four couples of photoanodes (eight photoanodes in total) were subjected to 10 ml of solution spray. The spray setup consisted of an ultrasonic spray head (Lechler, US1 308), set 30 cm above the substrates, which were placed on a hot plate, heated to 520°C (corresponding to a measured substrate surface temperature of about 400°C). An automatic syringe pump was used to deliver 1 ml of a solution to the spray head, every 30 s at a feeding rate of 12 ml per min (spray length of 5 s). The carrier gas (compressed air) flow, directing the spray to the substrates, was set to 15 ml per min. After spraying, the samples were cooled down in air to room temperature on the hot plate (turned off). The deposition rate for this process is known to be 1.5±0.3 nm of hematite per ml,[1] which means that the bottom layer thickness is 7.5±1.5 nm and the top layer thickness is 15±3 nm. The dimensions of the FTO substrate are 12.5 × 30 mm², 12.5 × 22 mm² of which are covered with hematite. The sample was part of a study that examined the effect of doping in the bottom and top hematite layers, as reported elsewhere.[1] However, the sample that was examined as a case study in this work featured two undoped hematite layers.

All measurements were conducted at ambient temperature (air condition 23°C) in alkaline aqueous solution (1M NaOH in deionized water). The photoanode was put in a so-called "cappuccino cell" as described in Ref. [2]. The potential *U* was adjusted versus a reference electrode. Basic working principles of reference electrodes and their application to PEC for water splitting cells can be found elsewhere.[3,4] In this study we used an Hg/HgO reference electrode in 1M NaOH aqueous solution, which is stable during long measurements in alkaline solutions since the internal electrolyte is also 1M NaOH, the same as the electrolyte in the PEC cell. The reference electrode has a potential of 0.930 V_{RHE}. Experiments conducted longer than one day revealed that commonly used Ag/AgCl reference electrodes can show a slow drift in potential of several 10's of mV, whereas Hg/HgO reference electrodes remained stable for several days (not shown here).

PEIS, IMPS and IMVS measurements and *J-U* curves were conducted via a Zahner Zennium electrochemical workstation equipped with a CIMPS system.[5] The light source was high power blue LED (Zahner KBR02) with a dominant wavelength of 449.13 nm (half width 13 nm) with a maximum intensity of 100 mW/cm² (based on manufacturer specifications). The frequency range for all the immittance measurements was 100 kHz to 100 mHz. The amplitude for the light intensity excitation for IMPS and IMVS measurements was 3.5 mW/cm² (corresponding to 10 mV excitation amplitude adjusted at the potentiostat controlling the LED) and 10 mV for PEIS. The duration for collecting each spectrum was 10 to 13 min.

S2. Light Intensity and Photon Flux

The photon flux, Φ, is defined as the number of photons per time period per unit area:

$$\Phi = \frac{\#\text{ of photons}}{t \cdot A}. \quad \text{(S1)}$$

The unit of Φ is $s^{-1}m^{-2}$ and it directly indicates the number of photons that arrive at the surface of the cell. Distinctions accounting for different wavelengths can be easily introduced by specifying a wavelength specific photon flux, $\Phi(\lambda)$. However, measurement devices usually work with the light intensity I as measured by a photodiode. I is the energy per time period per unit area:

$$I = \frac{E}{t \cdot A}. \quad \text{(S2)}$$

In order to convert a signal from Φ to I, the spectrum of the lamp is required. The lamp spectrum is either given as wavelength dependent flux Φ or in counts as arbitrary unit. For both cases the spectrum $\Phi_{lamp}(\lambda)$ has to be multiplied by hc/λ to obtain the (relative) energy of the emitted light intensity and normalized to mW/cm².

S3. Electrochemical Immittance (Impedance/ Admittance) Spectroscopy

General Introduction

Electrochemical impedance spectroscopy (EIS) is a powerful and well-established tool to investigate the dynamic processes that are responsible for losses during the operation of electrochemical systems. PEIS, IMPS and IMVS are related techniques that yield different types of photoelectrochemical immittance (i. e. impedance or admittance[6]) spectra, as introduced in equations (3), (4) and (5) of the article, and for which the same analysis tools can be applied.

The goal of immittance measurements is to probe the dynamic behavior of the system, which is determined by a number of physical, chemical or (photo)electrochemical processes with different time constants. Whereas static measurements can only measure the behavior of the sum of all the processes involved, impedance techniques can separate the overall resistance into contributions of different processes, provided that they show different time constants.

The measured quantity is $Z(\omega)$, the complex and frequency dependent resistance of the cell in a given operating point, consisting of the real part, $Z'(\omega)$, and the imaginary part, $Z''(\omega)$ for every measurement frequency ω:

$$Z(\omega) = Z'(\omega) + iZ''(\omega) \tag{S3}$$

Both the excitation and response signals have the general form

$$S(t) = S_0 + \hat{S} \cdot \sin(\omega t + \varphi_S), \tag{S4}$$

with S_0 being the bias value, $\hat{S}$, being the small signal AC amplitude of the sinusoidal signal with the phase angle φ_S. The impedance $Z(\omega)$ is calculated for each measurement frequency ω from the signals $U(t)$ and $J(t)$ that have the form of equation (S4),

$$Z(\omega) = \frac{U(\omega)}{J(\omega)} = \frac{\hat{U}}{\hat{I}} e^{i(\omega t(\varphi_U - \varphi_I))}. \tag{S5}$$

$U(\omega)$ and $J(\omega)$ are the Fourier coefficients of $U(t)$ and $J(t)$ with respect to ω, accounting only for the oscillating small signal component of the signals. The inverse value of $Z(\omega)$ is the admittance $Y(\omega)$:

$$Y(\omega) = Z(\omega)^{-1} = \frac{J(\omega)}{U(\omega)}. \tag{S6}$$

Impedance and admittance data can be analyzed with the same approaches and are summarized as immittance.[7,8] For very low frequencies, $Z(\omega)$ converges to one point on the real axis, the DC or internal resistance, $Z(0)$, as shown in Figure S1a (this is not the case for capacitive systems such as batteries or supercapacitors[9]). $Z(0)$ is the inverse slope of the *J-U* curve, because all dynamic processes have decayed and it is

$$Z(0) \stackrel{\text{def}}{=} Z(\omega = 0) = \frac{\mathrm{d}U}{\mathrm{d}J}|_{U_0}. \tag{S7}$$

Comparing $Z(0)$ and the slope of the *J-U* curve is an easy way to check consistency of static and dynamic measurements. If there is a mismatch, at least one of the measurement results is incorrect. For very high frequencies, $Z(\omega)$ converges to another point on the real axis, the so-called ohmic resistance, R_∞, because there are no polarization losses as the sluggish loss processes are not excited. Both $Z(0)$ and R_∞ are illustrated in Figure S1a. When comparing the impedance in Figure S1a with the admittance in Figure S1b, it becomes apparent that the diagrams are flipped over, in accordance with equation (6): lower absolute values are obtained for higher frequencies and the imaginary part $Z''(\omega)$ is positive for all ω (note that the Nyquist diagram for $Y(\omega)$ is usually plotted with a positive imaginary axis).

The admittance is rarely used in the literature but bares some advantages for displaying and fitting impedance data with $Z(\omega)\rightarrow\infty$ for $\omega\rightarrow\infty$ or to particularly analyze the high frequency behavior, as it is done for Mott-Schottky analysis.[10] Also the result of IMPS is displayed as admittance, as described in section 2.3 of the article. In some cases such as IMPS, it is preferable to depict the admittance, because $Z(\omega)\rightarrow\infty$ for

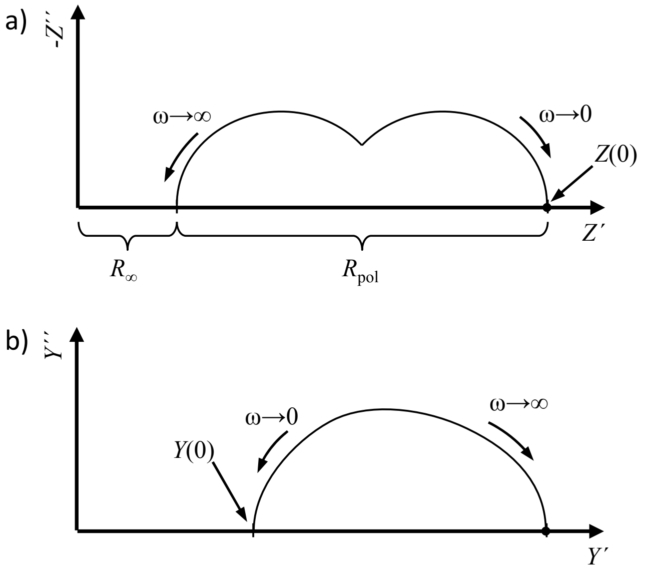

Figure S1: Schematic illustration of (a) impedance and (b) admittance spectra for an electrochemical system consisting of purely capacitive elements (such as RC elements).

$\omega\rightarrow\infty$, whereas $Y(\omega)\rightarrow 0$ for $\omega\rightarrow\infty$, which allows for a more intuitive diagram.

Generally, impedance and admittance are defined for the relation between current and voltage. In (photo)electrochemistry, the current is commonly substituted by the current density. However, other combinations of quantities are possible and it might be sensible to establish new forms of "impedances"[11] or "admittances", such as IMPS and IMVS. Following the definition in Ref. [11], the IMPS result is called photocurrent admittance. This definition complies with the concept that an impedance is the "cause" divided by the "effect".[11] In the case of IMVS this is a bit more difficult and here we rely on Ref. [12], where IMVS is defined as photovoltage impedance based on considerations of "across" (photovoltage) and "through" quantities (light flux).

Kramers-Kronig Test for Immittance Data

The Kramers-Kronig (KK) test is a powerful and well-established tool to test the quality of immittance spectra.[13] It is based on the KK relations that relate the real part of the immittance spectrum of a linear, time-invariant and causal system to its imaginary part and vice versa.[14] KK tests, such as "Lin-KK",[14] yield residual plots, that indicate the deviation of the measured spectrum from a perfectly KK compliant spectrum that fulfills the criteria of linearity, time-invariance and causality. In principle, it can be stated that if the residuals are small and randomly distributed, the spectrum is valid. Randomly distributed residuals show that a certain measurement noise is present during measurement, but the KK relations (therefore the abovementioned criteria) are basically satisfied. Systematically distributed residuals account for a violation of the criteria for the KK relations.

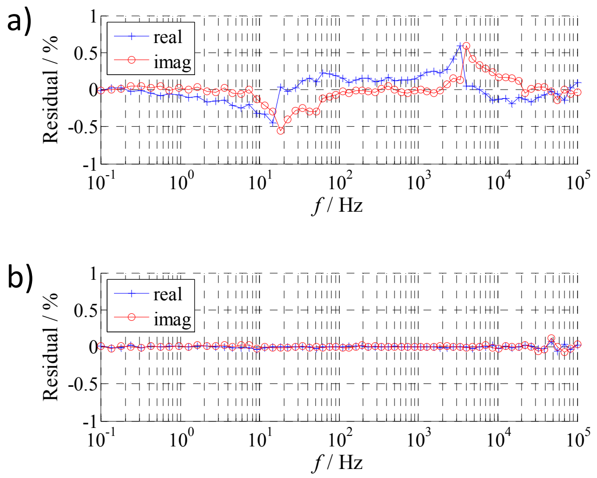

Figure S2. KK residuals calculated by "Lin-KK" for (a) the PEIS spectrum shown in Figure 4a of the article and (b) the IMVS spectrum shown in Figure 4b.

Figure S2 shows the KK residuals for the measured PEIS, IMPS and IMVS calculated by "Lin-KK".

It can be observed that the PEIS in Figure S2a shows some minor deviations around 10-20 and 3000-4000 Hz but the overall data quality is very good (residuals < 0.5%, for the most part). The residuals are so small that they probably originate from the measurement device itself, for example due to a switch in measurement ranges during measurement. The KK residuals for the IMVS measurement (Figure S2b) are negligibly small indicating a high quality measurement.

While the KK residuals for IMPS ($Y_{pc}(\omega)$) cannot be calculated directly, the very low residuals for PEIS and IMVS imply good quality for IMPS via equation (6).

The Effect of the Series Resistance on Immittance

A puzzling effect encountered in IMPS measurements was described by Ref. [15]. Apparently the time constant of the large semicircle in IMPS measurements ($Y_{pc}^{+}(\omega)$) changes if the series resistance of the PEC cell changes. In this section we will give a simple explanation for this effect.

We consider a parallel connection of a resistor R_1 and a capacitor C_1, an RC element, in series with a resistor R_∞. The characteristic frequency $2\pi f_c$ is the frequency for which the magnitude of $Z''(\omega)$ shows its maximum, $1/R_1C_1$. However, when calculating the frequency for which $Y''(\omega)$ shows its maximum in magnitude, we obtain

$$2\pi f_c = \frac{1}{R_1 C_1} \cdot \frac{R_\infty + R_1}{R_\infty}. \qquad \text{(S8)}$$

Further assuming that the series resistance $R_\infty << R_1$, equation (8) can be simplified to

$$2\pi f_c = \frac{1}{R_1 C_1} \cdot \frac{R_1}{R_\infty} = \frac{1}{R_\infty C_1}, \qquad \text{(S9)}$$

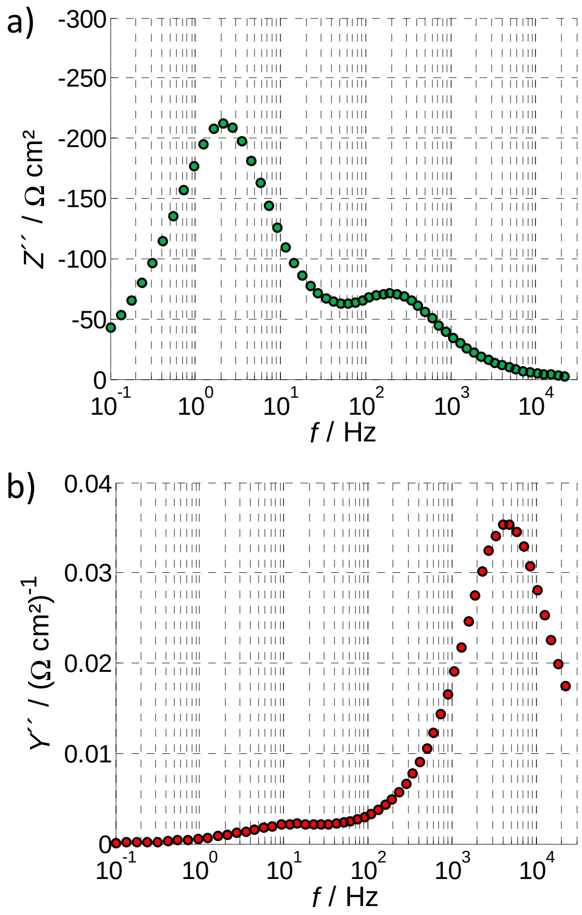

Figure S3. (a) Imaginary part of the impedance Z(ω) for the PEIS spectrum shown in Figure 4a; (b) imaginary part of the corresponding admittance $Y(\omega) = (Z(\omega))^{-1}$.

which means that it is considerably shifted. Such a shift is visible when comparing the imaginary parts of the impedance (Figure S3a) and admittance (Figure S3b) of the PEIS spectrum shown in Figure 4a of the article. The characteristic frequencies are shifted by about three decades. If we change equation (6) to

$$Y_{pc}(\omega) = \frac{Z_{pv}(\omega)}{Z(\omega)} = Z_{pv}(\omega) \cdot Y(\omega), \qquad \text{(S10)}$$

it is obvious that the characteristic frequencies of $Y_{pc}(\omega)$ is influenced by $Y(\omega)$ (equation (S10)), which directly depends on R_∞ (equation (S9)).

S4. Example Fits for Two Standard ECMs

As already mentioned in section 3.1, there is generally no unique solution for an ECM. In the example given here we fitted the PEIS spectrum shown in Figure 4a to both of the ECMs in Figure 6 (model 1 and 2, respectively). Subsequently, we replaced the two capacitors by constant phase elements (CPE) and the fits improved significantly (model 3 and 4, respectively). Table S1 shows the fit results obtained by commercial software for all circuit elements plus the ΣX^2 value, which is an indicator of the "goodness of the fit".

It can be observed that:

- The ECMs in Figure 6 lead to the same "goodness of fit" (ΣX^2) while producing slightly different values for R_1, R_2, C_1 and C_2). This is in agreement with Ref. [17] (see also section 3.1).
- Much better "goodness of fit" can be achieved by replacing the capacitors in Figure 6 by CPEs, which again leads to similar fit results for models 3 and 4 in terms of the parameter values and the "goodness of the fit".
- Acceptable fits for the hematite photoanode measured here (Figure 4a) are only possible with modifications of the ECMs, namely replacing all capacitors by CPEs as presented in Refs. [18] and [19].
- It can hardly be judged from these results, which of the ECM is more suitable to describe the behavior of hematite photoanode accurately.

Another important indicator to judge the quality of an ECM and the corresponding fit is the residual plot, which is often omitted, unfortunately. Similar to the KK test discussed in section S3, only small and randomly distributed residuals indicate appropriate fit results. Figure S4 shows the residuals for the fits compiled in Table S1.

It is most apparent that the residuals for all the fits to models with only capacitors show very large systematic deviations. The values of 10% are far beyond the limit that can be considered a good fit, even though the quality of the measurement was very good, as confirmed by the KK residuals in Figure S2a. Replacing the capacitors by CPEs yields an improvement by one order of magnitude as residuals are below 1% in Figure S4c and d. However, these two plots also show some oscillations – i. e. systematic deviations – below 10 Hz that indicate that the model does not perfectly describe the behavior of the photoanode. The residuals for the DRT spectra in Figure 8 are small also at low frequencies. The only deviations in Figure S4e are around 10-20 and 3000-4000 Hz, where also the KK residuals in Figure S2a showed some minor artefacts that might also be caused by measurement errors (see also section S3).

By this example we showed that it is quite difficult to produce good fit results even with high quality PEIS measurements. The ambiguity of two common ECMs for hematite photoanodes was also demonstrated plus the superior accuracy for the DRT calculation, which is the basis of our empirical analysis approach.

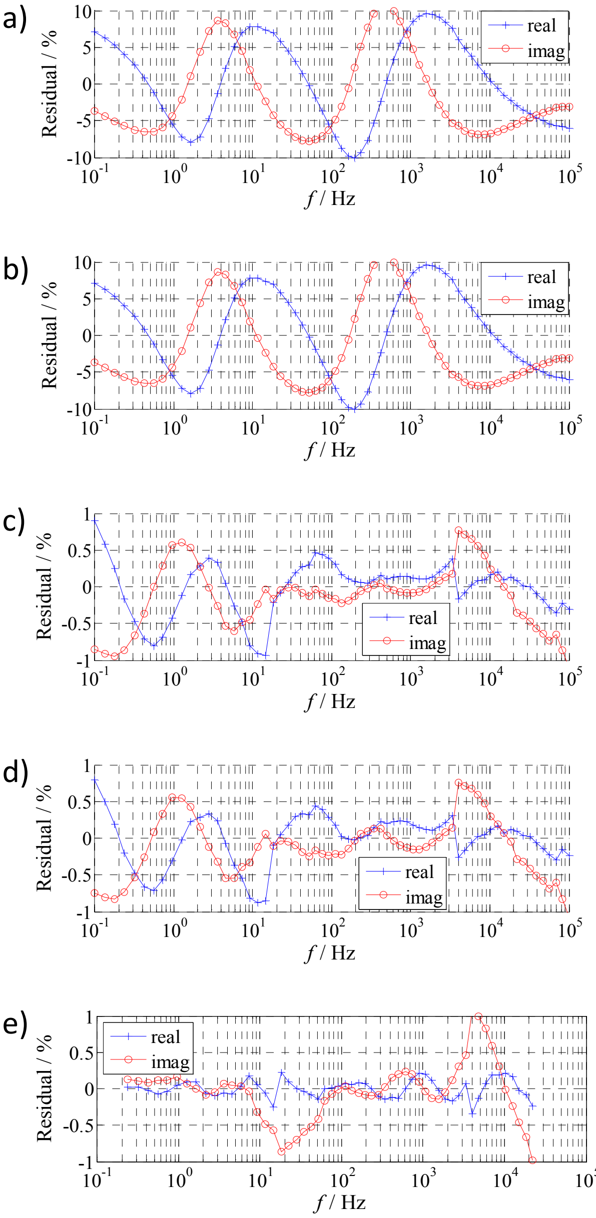

Figure S4. Residual plots for the fit results compiled in Table 1, (a) to (d) models 1-4, and (e) for the calculation of the DRT shown in Figure 8. Note the different scaling of the y-axis in (a) and (b) as compared to (c), (d) and (e).

Model	R_S	R_1	C_1	CPE_1	R_2	C_2	CPE_2	ΣX^2
**1**	12.1	153	3.86	-	466	125	-	1.75
**2**	12.1	144	3.98	-	475	126	-	1.75
**3**	11.4	160	-	8.67 (0.91)	511	-	210 (0.86)	$6.99 \cdot 10^{-3}$
**4**	11.3	143	-	9.21 (0.91)	530	-	213 (0.86)	$6.06 \cdot 10^{-3}$

Table S1. Fit results obtained for fitting the PEIS spectrum from Figure 4a to both of the ECMs shown in Figure 6a (model 1) and Figure 6b (model 2), and replacing the capacitors by constant phase elements. The names of the elements in this table are taken from the brackets in Figure 6. All resistances are given in Ωcm² and capacitors and constant phase elements (CPE)[16] are given in µF/cm² (for the CPEs the exponent is provided in brackets).

S5. Linear Curve Fitting

Static curves such as the *J-I* curve can be fitted with a linear curve fitting approach. The fit problem is rewritten as a system of linear equations.

For example let us assume a curve fitted with a polynomial function of the order n,

$$y = c_n x^n + c_{n-1} x^{n-1} + \cdots + c_1 x^0 \,. \quad \text{(S11)}$$

The fit problem can be written as

$$A \cdot c - b = 0, \quad \text{(S12)}$$

with the goal to find the appropriate coefficient vector *c*, which is defined as

$$c^T = [c_n \quad \cdots \quad c_1]. \quad \text{(S13)}$$

Any dataset that adds information to the fit problem yields a row a^{T} in the matrix A (n columns) and an element in the vector b. A static measurement point ($x, f(x) = y$) yields:

$$a^T = [x^n \quad \cdots \quad x^0], b = y \,. \quad \text{(S14)}$$

From immittance data, the derivatives in a measurement point can be obtained for $\omega \to 0$, accounting for the local slopes in the *J-U* or *J-I* curves. Immittance data ($x, f'(x) = y'$) yields:

$$a^T = [n \cdot x^{n-1} \quad (n-1) \cdot x^{n-2} \quad \cdots \quad x^0 \quad 0], b = y' \,. \quad \text{(S15)}$$

In order to solve the linear system in equation (S12), we apply the method "linsolve" in Matlab that uses LU factorization. The order n should be chosen as small as possible in order to limit the degrees of freedom and to obtain a smooth curve. On the other hand it should account for the characteristic features of the curve. For *J-I* curves, an order of $n = 2$ seems to be reasonable for small errors and good agreement with the static and immittance measurements.

Other static curves such as *J-U* curves exhibit plateaus and other features than can hardly be represented by polynoms. For fitting these curves, a nonlinear curve fitting approach is required. The Matlab method "fit" is well-suited for this purpose.

References

1. T. Hisatomi, H. Dotan, M. Stefik, K. Sivula, A. Rothschild, M. Grätzel and N. Mathews, *Adv. Mater.*, 2012, **24**(20), 2699.
2. K. D. Malviya, H. Dotan, K. R. Yoon, I.-D. Kim and A. Rothschild, *J. Mater. Res.*, 2015, 1.
3. H. Dotan, K. Sivula, M. Grätzel, A. Rothschild and S. C. Warren, *Energy Environ. Sci.*, 2011, **4**(3), 958.
4. A. J. Bard and L. R. Faulkner, *Electrochemical methods: Fundamentals and applications*, Wiley, New York NY u.a., 2001.
5. http://www.zahner.de/pdf/CIMPS.pdf.
6. B. Boukamp, *Solid State Ionics*, 1986, **20**(1), 31.
7. J. R. Macdonald and E. Tuncer, *J. Electroanal. Chem.*, 2007, **602**(2), 255.
8. B. Boukamp, *Solid State Ionics*, 1986, **18-19**, 136.
9. J. Illig, M. Ender, T. Chrobak, J. P. Schmidt, D. Klotz and E. Ivers-Tiffee, *J. Electrochem. Soc.*, 2012, **159**(7), A952-A960.
10. R. Franking, L. Li, M. A. Lukowski, F. Meng, Y. Tan, R. J. Hamers and S. Jin, *Energy Environ. Sci.*, 2013, **6**(2), 500.
11. E. Barsoukov, ed., *Impedance spectroscopy: Theory, experiment, and applications*, Wiley-Interscience, Hoboken NJ u.a., 2005.
12. L. M. Peter and D. Vanmaekelbergh in *Advances in Electrochemical Science and Engineering*, ed. R. C. Alkire and D. M. Kolb, Wiley-VCH Verlag GmbH, Weinheim, Germany, 1999, p 77.
13. B. A. Boukamp, *J. Electrochem. Soc.*, 1995, **142**(6), 1885.
14. M. Schönleber, D. Klotz and E. Ivers-Tiffée, *Electrochim. Acta*, 2014, **131**, 20.
15. W. Liu, L. Hu, S. Dai, L. Guo, N. Jiang and D. Kou, *Electrochim. Acta*, 2010, **55**(7), 2338.
16. M. R. Shoar Abouzari, F. Berkemeier, G. Schmitz and D. Wilmer, *Solid State Ionics*, 2009, **180**(14-16), 922.
17. J. R. Macdonald, *Electrochim. Acta*, 1990, **35**(10), 1483.
18. B. Klahr, S. Gimenez, F. Fabregat-Santiago, T. Hamann and J. Bisquert, *J. Am. Chem. Soc.*, 2012, **134**(9), 4294.
19. T. Lopes, L. Andrade, F. Le Formal, M. Gratzel, K. Sivula and A. Mendes, *Phys. Chem. Chem. Phys.*, 2014, **16**(31), 16515.
